# Protonation States of Proton-Sensing Glutamate Residues in Transporter Sialin

**DOI:** 10.3390/ijms27104629

**Published:** 2026-05-21

**Authors:** Eric Wooten, Nara L. Chon, Muhamadjon Dzhalolov, Hongjin Zheng, Hai Lin

**Affiliations:** 1 Department of Chemistry, University of Colorado Denver, Denver, CO 80217, USA; eric.wooten@ucdenver.edu (E.W.); nara.chon@ucdenver.edu (N.L.C.); muhamadjon.dzhalolov@ucdenver.edu (M.D.); 2 Department of Microbiology, University of Alabama at Birmingham, Birmingham, AL 35294, USA; 3 Center of Advanced Computational Molecular Sciences, University of Colorado Denver, Denver, CO 80217, USA

**Keywords:** arginine-to-lysine mutation, continuum solvation model, salt bridge, sialic acid, steered molecular dynamics

## Abstract

Sialic acids are a diverse class of widely distributed monosaccharides that are engaged in a wide range of biological processes. Sialin, a sialic acid/proton symporter, transports sialic acid across membranes between the lysosomal lumen and cytosol, playing a critical role in sialin metabolism. Taking advantage of recently published experimental structures of sialin, we report here the first computational study that probes the molecular mechanism of ligand transport through sialin, which is yet to be fully understood. In particular, we carry out steered molecular dynamics simulations of the transport of *N*-acetylneuraminic acid, the most widely spread natural derivative of sialic acids, through sialin with two key glutamate residues (E171 and E175) in various protonation states. The previously proposed model is refined with enriched atomistic details from this study for the cotransport of sialic acid and proton. With additional quantum calculations, our data suggest a possible explanation for why mutation R168A retains most of the transport activities, but R168K does not.

## 1. Introduction

Sialic acids are a diverse group of nine-carbon monosaccharides that are ubiquitously present in animals and some bacterial species [1]. They are engaged in a wide spectrum of biological processes, including immune responses and the control of protein stability [2,3]. In particular, sialic acids are related to many diseases in humans, such as the Salla disease [4,5], cancer [6], immunological disorders [7], and type 2 diabetes [8]. Thus, it is important to understand the synthesis, distribution, and degradation of sialic acids.

**Figure 1:**
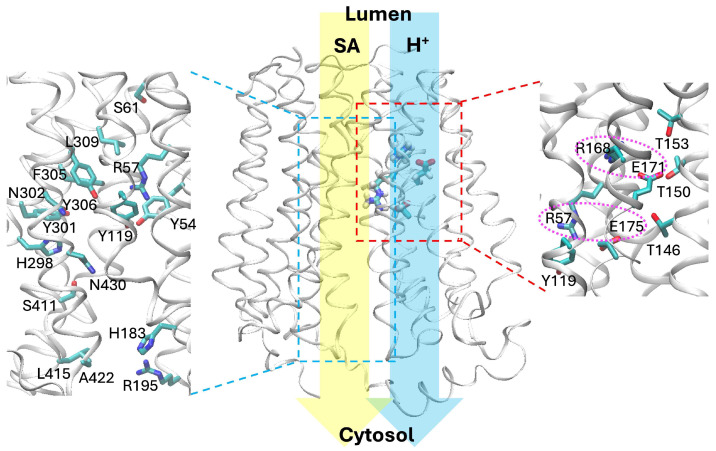
Overall protein architecture of sialin (PDB 8DWI). The protein is shown as helices and loops in white, and residues as sticks (color code: C, cyan; N, blue; O, red). Four residues, R57, R168, E171, and E175, are displayed in the central panel. The blue and yellow vertical arrows indicate the cotransport of proton (H^+^) and sialic acid (SA) from the lumen (outside) to the cytosol (inside), respectively. The inset on the left (indicated by the blue dashed rectangle) exhibits the critical residues along the SA translocation path in the inward-facing conformation. The inset on the right (indicated by the red dashed rectangle) shows the residues in the tunnel that connects E171 and E175 to the lumen, with the two salt bridges marked by dotted cycles.

Sialin, a member of the solute carrier 17 (SLC17) family of transport proteins [9], plays a critical role in sialic acid metabolism. Sialin transports sialic acids across membranes between the lysosomal lumen and cytosol, although it is also involved in the translocation of many other organic anions such as glutamate, aspartate, nitrate, and phosphate [10]. Published in 2023 by Zheng and coworkers [11], the first high-resolution (3.4 Å) experimental structure of wild-type (WT) sialin (PDB 8DWI) was obtained through cryogenic electron microscopy (cryo-EM). As shown in Figure 1, sialin contains 12 transmembrane helices (TM1-TM12) in an overall architecture resembling the canonical fold for the major facilitator superfamily. The N-domain is made of TM1–TM6, and the C-domain of TM7–TM12. TM6 and TM7 are connected by a cytosolic loop (L7), which shows a well-defined small cytosolic helix (Cyt) formed by residues E264-N272 right beneath the N-domain. This structure corresponds to an inward-facing partially open conformation, where a putative ligand binding-site pocket is located along the central translocation pathway between the N- and C-domains. The pocket is embraced by residues Y54, R57, Y119, H298, Y301, N302, F305, Y306, S411, and N430, which presumably stabilize sialic acids through hydrogen bonds and salt bridges. Docking calculations were able to fit a natural ligand, *N*-acetylneuraminic acid (Neu5Ac), in this pocket. The structure also reveals a small tunnel in the N-domain that connects the lumen with two strictly conserved glutamate residues (E171 and E175) deep in the protein center. Mutagenesis experiments confirmed that both E171 and E175 are critical for sialin operation, likely in proton sensing and/or translocation. The cryo-EM structure was further compared with a structure AF-Q9NRA2-F1 predicted by AlphaFold [12], but the two structures do not overlap well. It was speculated that the AlphaFold structure is in a partially outward-opening conformation, but whether it is physiologically relevant remains to be seen.

More recently, a second set of cryo-EM structures was reported for sialin at up to 2.8 Å resolution in various experimental conditions by Schmiege et al. [13]. In the apo state, the WT protein under both pH = 5.0 and pH = 7.5 conditions exhibits almost identical conformations as the one reported by Zheng and coworkers [11], with a root-mean-square-deviation (RMSD) value of ~0.5 Å for the backbones. Two mutants, S61A and R168K, both at pH = 7.5, were captured in the outward-opening conformations. In both mutants, the interactions seen in the WT between the R168 side chain and the backbone carbonyl group of R57 are disrupted, and R/K168 forms a salt bridge with E171. Also disrupted is the hydrogen bond between the hydroxyl group of the S61 side chain and the carbonyl group of the L309 backbone. These observations suggested that the protein conformational changes may be modulated by the interaction network in the luminal leaflet. Schmiege et al. [13] further determined the WT structures in complex with a ligand, *N*-acetylaspartylglutamate (NAAG) and a non-competitive inhibitor, fluorenylmethyloxycarbonyl chloride, respectively. Both structures were obtained at pH = 7.5 and were found to be in the inward-opening conformations. The authors performed 100 ns molecular dynamics (MD) simulations at the molecular-mechanics (MM) level to equilibrate the NAAG-bound model embedded in a lipid bilayer of 1-Palmitoyl-2-oleoyl-*D*-glycero-1-phosphatidylcholine (POPC), and they found that NAAG left the binding site and diffused into the cytosol quickly (after ~50 ns).

**Figure 2:**
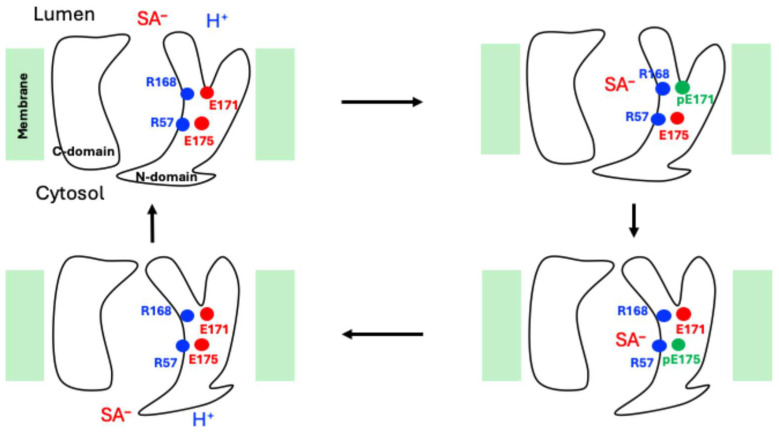
Proposed proton/sialic acid cotransport model of sialin [11]. The charges of proton and sialic acid (SA) are explicitly displayed, and pE171 and pE175 denote the protonated E171 and protonated E175, respectively.

These structural studies shed light on the transport mechanism of sialic acids by sialin. The proposed proton/sialic acid cotransport model by Zheng and coworkers [11] (Figure 2) starts the cycle with sialin in the outward-opening conformation, where R168 forms a salt bridge with E171 and R57 interacts with E175. The binding of sialic acid disrupts the R168-E171 interactions: sialic acid takes R168, freeing E171 to accept a proton from the lumen solution. Moving down along the translocation path, sialic acid similarly takes R57, allowing the proton to be relayed between the protonated E171 (pE171) and E175. After releasing the proton, E171 returns to facing R168. Meanwhile, sialic acid travels further down the path. In the inward-opening state, the protonated E175 (pE175) releases the proton to the cytosol and, before rebinding R57, prompts the sialic acid to be released into the cytosol. Finally, sialin is reset to the outward-opening conformation, ready for the next cycle. The model by Schmiege et al. [13] is essentially the same.

The above model is consistent with many experimental findings, such as the significantly reduced ligand transport in mutants R57A, E171Q, and E175Q. However, the atomistic details are yet to be fully comprehended, especially regarding the coupling between the SA and proton that leads to the 1:1 stoichiometry of cotransport. For example, it remains to be elucidated how the proton from the lumen is uptaken by E171, which is located deep in the protein. Also, how does the binding of sialic acid influence the protonation of E171 and E175, and vice versa? It is puzzling that, while the structures of the R168K and S61A mutations suggest that the interactions between R168 and E171 are crucial for sialin to achieve outward opening [13], mutant R168A, where the R168-E171 salt bridge is lost, retains most of the ligand transport function [11]. This raises the question: How important is the R168-E171 salt bridge in the alternations of the protein conformation between the inward-opening and outward-opening states? Also, while R57 forms a salt bridge with E175, why do mutants R57A [11] and R57K [13] become seriously dysfunctional?

Clearly, there remain a lot of questions to be answered. To satisfactorily address these questions requires significant efforts from both experimental and computational researchers. Here, we report the first computational study that simulates the translocation of sialic acid through sialin by steered-MD (SMD) simulations with trajectories of a combined simulation time of more than 5 μs. As a non-equilibrium simulation technique, SMD has limitations and may cause artifacts. Nevertheless, when used with care, it is a convenient and helpful tool to accelerate many slow processes that are currently still beyond reach in atomistic MD simulations. In this work, the goal of our SMD simulations is to explore if and how the various protonation states of E171 and E175 may potentially affect the protein undergoing conformational change during ligand transport. Our data suggest that protonating E171 and E175 disrupt the salt bridges with R168 and R57, respectively, and that the changes may make it easier for sialin to realize the outward-facing conformations. We also carried out additional continuum solvation quantum model calculations for the salt bridges R168-E171 in the WT and K168-E171 in the R168K mutant, and the results suggest a possible explanation for why this mutant is dysfunctional. Based on the results, the previously proposed proton/sialic acid cotransport model is refined with additional atomic details.

## 2. Results

### 2.1. Model Equilibrations

#### 2.1.1. Equilibrated Protein Conformations

Three atomistic models of WT sialin were equilibrated, which were named “dp” (stands for deprotonated E171 and E175), “p171” (stands for protonated E171 and deprotonated E175), and “p175” (stands for protonated E175 and deprotonated E171). The generated trajectories are denoted “dpeq”, “p171eq”, and “p175eq”, respectively, where “eq” stands for equilibration. Inspections of the equilibration trajectories revealed that the N-terminal loop (A32-S37) and the cytosol loop (V248-P281, including the short cytosol helix Cyt) were highly flexible, while the transmembrane helices (TM1 to TM12) were more stable. This is confirmed by the plots of the backbone RMSD of individual residues, averaged over simulation time (Figure S1 in the Supporting Information). The plots are qualitatively similar for all three models, showing very large RMSD (>6 Å) for the N-terminal and the cytosol loops, while small RMSD (<2 Å) for most residues in the transmembrane helices. Most inter-TM loops and many TM residues near these loops are also quite flexible (RMSD > 4 Å), such as those connecting TM4 and TM5 (denoted TM4/5) in all models, TM8/9 in dp, TM9/10 in p175, and TM11/12 in both p171 and p175. These flexible regions are all located on the cytosol side in dp, but they are found on both the cytosol and lumen sides in the other two models.

These results imply overall similar protein conformations, as echoed by the overlays of representative equilibrated geometries on 8DWI, which provide visual depictions of how well these protein structures superimpose with each other (Figure S2 in the Supporting Information). Small (<2 Å) average backbone RMSD with respect to 8DWI were found for these equilibrated geometries: 1.5 Å (dp), 1.9 Å (p171), and 1.8 Å (p175), respectively (Table S3 in the Supporting Information). Note that when assessing protein conformations, unless explicitly indicated, we excluded the N-terminal and cytosol loops from the RMSD average, since their movements are not relevant. Overall, the equilibrated proteins do not differ significantly from 8DWI, based on which our models were constructed.

#### 2.1.2. Pore Opening

**Figure 3:**
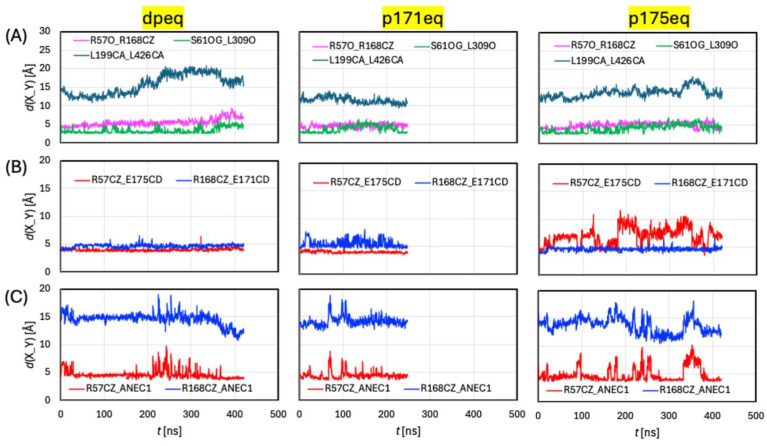
Distances over simulation time (t) during equilibrations: (**A**) Distances from S61Oγ to L309O, from R168Cζ to R57O, from L199Cα to L426Cα. (**B**) Distances from R57Cζ to E175Cδ and from R168Cζ to E171Cδ. (**C**) Distances from ligand Neu5Ac (ANE) C1 atom to R57Cζ and R168Cζ, respectively.

Inspired by Schmiege et al. [13], we attempted two metrics to gauge the outward opening of the pore: (1) the distance *d*(S61Oγ-L309O) between the S61 side chain hydroxyl group and the L309 backbone carbonyl group, and (2) the distance *d*(R168Cζ-R57O) between the R168 side chain guanidino group and the R57 backbone carbonyl group. Here, we plot these distances against simulation time (t) in Figure 3A. In structure 8DWI, *d*(S61Oγ-L309O) = 3.6 Å, and *d*(R168Cζ-R57O) = 4.7 Å. During the equilibrations, they varied between 3 and 5 Å and between 4 and 6 Å, respectively, except that *d*(R168Cζ-R57O) increased to ~8 Å toward the end of the dp equilibration. The short distance of *d*(S61Oγ-L309O) and its small (~2 Å) fluctuations are consistent with the side chain–backbone interaction not changing appreciably during the equilibration and that the protein’s lumen opening remained narrow. The larger variations of ~4 Å in *d*(R168Cζ-R57O) observed in dp are a bit puzzling, and we suspect that this distance may be a less effective metric to gauge the pore’s outward opening.

What about the protein’s opening toward the cytosol? Trajectory inspections revealed subtle variations between the models, even though they all resembled the inward-opening 8DWI. Specifically, the cytosol end of TM5 (R195-G205) in p171 bent toward TM11 with G205 as the pivot point. The bending of TM5 was only ~14°, but it allowed the association of the hydrophobic side chains of L199 and L426 that effectively closed the pore toward the cytosol in p171 (lower panels in Figure S2 in the Supporting Information). This is reflected by the plots of distance *d*(L199Cα-L426Cα) in Figure 3A, which measures ~15 Å in 8DWI. Over the equilibrations, *d*(L199Cα-L426Cα) increased to ~17 Å (fully open) in dp, dropped to ~10 Å (closed) in p171, and remained largely at ~14 Å (partially open) in p175. Such structural changes, which are the biggest for p171 and smallest for p175, are consistent with backbone RMSD average over R195-G205 for the representative geometries of dp (2.3 Å), p171 (3.9 Å), and p175 (1.8 Å) with respect to 8DWI.

**Figure 4:**
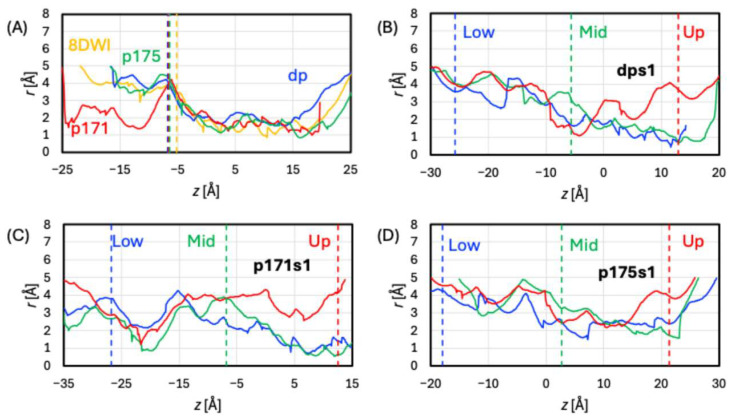
Pore radius along the transport path for representative geometries extracted from the (**A**) equilibrated, (**B**) dps1, (**C**) p171s1, and (**D**) p175s1 trajectories. Results for the experimental protein structure (PDB 8DWI) are shown in panel (**A**). For the SMD trajectories, results for all Up, Mid, and Low geometries are shown, while only the Mid geometries are available for the equilibration trajectories and the experimental structure. A vertical dashed line indicates the z coordinate of the C6 atom in the given geometry.

Further insights are provided by HOLE calculations for the representative geometries. Such a snapshot extracted from the trajectories will be denoted “Up” if Neu5Ac is located in the upper section of the path crossing the membrane, “Mid” if Neu5Ac is in the middle of the protein near the binding site, or “Low” if Neu5Ac is in the lower section of the path crossing the membrane. The Up and Low geometries correspond approximately to the moments when the ligand entered and exited the pore, respectively. For the equilibration runs, only the Mid geometries were available. The pores are depicted in Figure S3 in the Supporting Information. The pore radii (*r*) along the transport path are plotted in Figure 4A, where the locations of Neu5Ac identified by the *z* coordinate of its C6 atom are indicated by vertical dashed lines (at *z* = –5 or –6 Å, depending on the given model). Notably, over a long section of the pore (from *z* = –6 to 15 Å), the radii are similar for 8DWI and all equilibrated models. The overall shape of Neu5Ac is roughly an ellipsoid, with the length of the semi-axes *a*~*b*~9 Å and *c*~6 Å. Thus, the pore radius must be at least 3 Å to allow the ligand to push through. Apparently, the path between *z* = 0 and 15 Å is too narrow (*r* ≤ ~2 Å) for Neu5Ac to pass, thus preventing the sialic acid from freely diffusing through. The pore sections below the location of the ligand (*z* < –6 Å) are sufficiently wide in 8DWI, dp, and p175 but too narrow in p171.

We note that, in the outward-facing AlphaFold structure AF-Q9NRA2-F1 reported by Zheng and coworkers, the translocation path on the cytosolic side is entirely closed by H183, R195, L415, and L199 [11]. Although the inward-closed/outward-closed p171 and inward-closed/outward-opening AF-Q9NRA2-F1 have different overall conformations, the inward-closing of the pore seems to share some similarity in the involved residues.

#### 2.1.3. Salt Bridges R57-E175 and R168-E171

The four residues R57, R168, E171, and E175 formed two salt bridges: R57-E175 and R186-E171, as depicted in Figure 1, dotted ellipses. It has been proposed that the protonation of E171 or E175 by a proton from the lumen solution will lead to the disruption of the corresponding salt bridge, freeing the arginine to interact with the sialic acid in the pore. Here, the stabilities of the two salt bridges during the equilibrations are assessed by the distances *d*(R57Cζ-E175Cδ) and *d*(R168Cζ-E171Cδ), which are 4.5 and 5.0 Å in structure 8DWI, respectively. Note that the salt bridge distances were measured between the heavy atoms closest to the *charge center* of the involved side chain functional groups, e.g., the Cζ atom of arginine (for the guanidium cation), the Cδ atom of glutamate (for the carboxylate anion), and the Nζ atom of lysine (for the protonated amino group). These choices minimize the impacts due to the side chain rotations, which can swap the nitrogen and/or oxygen of a functional group participating in a salt bridge during a simulation. Such choices have been used previously by other researchers [14,15]. With such choices, we would consider a salt bridge to be (i) stable if the distance remained <5.5 Å for the Arg-Glu and <5.0 Å for Glu-Lys pairs, (ii) completely broken if the distance is >8.0 Å for the Arg-Glu and >7.5 Å for Glu-Lys pairs, and (iii) unstable if in between.

As can be seen from Figure 3B, both salt bridges were highly stable in dp. This was confirmed by the occupancy percentage of the salt bridge (summarized in Table S4 in the Supporting Information), which showed that both bridges were stable >99% of the time. In p171, the R168-E171 interaction was weakened, with *d*(R168Cζ-E171Cδ) fluctuating frequently between 5 and 7 Å, but the pair managed to hold together (stable ~65% of the time and unstable ~35% of the time). The biggest disruptions were observed for R57-E175 in p175, which was stable for only ~20% of the time and even broke for more than 25% of the time. The glutamate protonation had profound impacts on the salt bridges.

#### 2.1.4. Ligand Position and Orientation

Once the equilibrations started, the binding site was quickly filled with water molecules from the cytosol solution, and the ligand became well hydrated. Inspections of the trajectories revealed that Neu5Ac was very flexible in such an environment, rotating frequently, but staying near the binding site. This is reflected by the plots of the *z* coordinates of its C1 and C6 atoms against simulation time in Figure S4 in the Supporting Information. Very close (always within 1 Å) to the ligand’s center of mass (COM), C6 is a good indicator for the location of the ligand. In the docked geometry, *z*(C6) is ~–5 Å, while it fluctuated between –5 and –7 Å during model equilibrations, confirming that the ligand never moved far from the binding site.

The orientation of the Neu5Ac can be described by the polar angle *θ* (0° ≤ *θ* ≤ 180°) for the vector from COM to C1 (Figures S4C in the Supporting Information). The ligand’s carboxyl group points upward toward the lumen when *θ* < 90°, points downward toward cytosol when *θ* > 90°, and stays perpendicular to the *x*-*y* plane when *θ* = 90°. Because C6 is very close to COM, the orientation can also be inferred from the relative position of C1 in the carboxyl group to C6 (or COM): If *z*(C1) > *z*(C6), the carboxyl group is pointing upward, while *z*(C1) < *z*(C6) indicates the opposite. In the docking calculations, the ligand carboxyl group established a salt bridge with the side chain of R57. This salt bridge seemed quite stable (Figure 3C), as the distance between the C1 and R57Cζ atoms stayed mostly at ~5 Å, despite sometimes going above 7 Å, especially in p175. As shown in Table S5 in the Supporting Information, the stable occupancy time was ~80% or higher in all trajectories. Because R57 is located near the top of the binding site, it is not surprising that the ligand was mostly upward pointing during the equilibrations. On the other hand, the distance from C1 to R168 Cζ was very long (>10 Å), suggesting no salt bridge interactions between R168 and the ligand (Figure 3C and Table S5 in the Supporting Information).

### 2.2. Steered MD Simulations

For each of the three models, we have generated three independent SMD trajectories, which are carefully inspected and analyzed. The nine trajectories are named “dps1”, “dps2”, “dps3”, “p171s1”, … “p175s3”, respectively, where “s1”, “s2”, and “s3” denote the first, second, and third SMD runs, respectively. A summary of all SMD trajectories is given in Table S2 in the Supporting Information. For brevity and without loss of generality, the s1 trajectory of each model will be presented and discussed in detail, followed by the comparisons of s1 with the other two (s2 and s3) trajectories. Additional details of the s2 and s3 trajectories are provided in Figures S10–S21 in the Supporting Information.

#### 2.2.1. Overview of SMD Trajectories

As can be seen in Figure S6A in the Supporting Information, Neu5Ac migrated steadily through the pore in all s1 trajectories at an approximate speed of ~0.1 Å/ns, the same as the pulling speed of the reference point. This was also found for all s2 and s3 trajectories in this work, implying that the pulling speed was likely sufficiently slow so that the protein could adapt, at least locally, to enable a smooth translocation of the ligand.

Inspections of all trajectories revealed that the ligand was partly hydrated throughout its journey, more so in the lower than in the upper section of the path. The ligand was rather flexible in such an environment, rotating frequently and varying orientations from time to time. This is reflected by the relative position of C1 in the ligand carboxyl group to C6 (or COM), which is exemplified for all s1 trajectories in Figure S6B in the Supporting Information.

**Figure 5:**
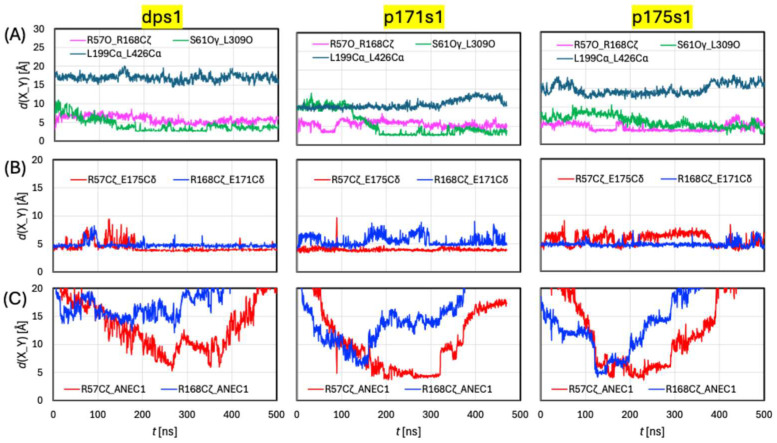
Key geometric distances over simulation time (t) in the s1 trajectories. (**A**) Distances from S61Oγ to L309O, from R168Cζ to R57O, from L199Cα to L426Cα. (**B**) Distances from R57Cζ to E175Cδ and from R168Cζ to E171Cδ. (**C**) Distances from ligand Neu5Ac (ANE) C1 atom to R57Cζ and R168Cζ, respectively.

To pinpoint the impacts of different protonation states of E171 and E175 between the three models, we assess how key geometric distances evolved over simulation time. These distances are plotted in Figure 5 using the s1 trajectories as examples. Also, the representative geometries are overlayed with 8DWI in Figure S7 in the Supporting Information. The transport paths of these geometries are depicted in Figure S8 in the Supporting Information, with the radii along the path plotted in Figure 4B–D.

#### 2.2.2. Deprotonated E171 and E175

Both E171 and E175 were deprotonated in the dp model. In dps1, the ligand entered the pore crossing the membrane at *t* ~ 30 ns, navigated through the upper section of the pore, passed L309 at ~200 ns into the lower section of the pore, and exited the pore crossing the membrane again at ~460 ns.

The pore opened modestly outward during ligand uptake, an event captured by the plot of distance *d*(S61Oγ-L309O) in Figure 5A. Analysis of the model equilibrations in Section 3.1 above showed that, when the pore is outward-closed, *d*(S61Oγ-L309O) fluctuated between 3 and 5 Å. Here, *d*(S61Oγ-L309O) started at ~9 Å, reduced to 5 Å by 100 ns, and remained at 3–5 Å until the end. On the other hand, *d*(R168Cζ-R57O) varied between 4 and 8 Å without displaying a strong trend of increase or decrease during the simulation, a feature repeated in the other SMD simulations. The brief pore opening was made possible by modest conformational changes in the lumen ends of TM1, TM7/8, and TM9/10 at the beginning of the trajectory, which were then closed for the rest of the time. This is evident from the backbone RMSD plots of individual residues in Figure S5A in the Supporting Information. The lower section of the path remained essentially the same during the simulations, in harmony with the inward-opening conformation of the equilibrated dp, and *d*(L199Cα-L426Cα) saw little change.

As expected, the two salt bridges R57-E175 and R168-E171 were very stable (>87% of the time) despite minor perturbations between 70 and 100 ns (Figure 5B) when the ligand traveled through the upper section of the pore. At this stage, the ligand was still quite far (>15 Å) away from R57 and R168 (Figure 5C) and thus did not interact directly with either arginine. The perturbations mainly came from interactions between the glutamate carboxyl groups and nearby hydroxyl groups of Y54 and Y119 (for E175) and T146 and T150 (for E171). Migrating through the protein, the ligand did not interact with R168 at all but formed a salt bridge with R57 three times after it entered the lower section of the pore, although the salt bridge lasted only briefly (~10 ns) each time. The disruptions of the salt bridge were due to rotations and reorientations of the ligand.

The representative geometries are overall similar to 8DWI, with the backbone RMSD of 2.0, 1.8, and 1.9 Å for the Up, Mid, and Low geometries, respectively (column 5 in Table S3). Given the modest outward-opening of the pore in the Up geometry, local side chain movements of the pore residues must be important in assisting the ligand transport. The pore’s lower section was wide open in all three geometries. The upper section of the pore was closed in the Mid and Low geometries but open near the entrance in the Up geometry. In the Up geometry, the ligand was prevented from freely diffusing through by a bottleneck (*r* ~ 1 Å) near the binding site, which was formed by clustering side chains of S54, R57, Y119, Q123, and E175.

The dps2 trajectory exhibited some interesting differences from dps1. First, there was no apparent pore outward-opening due to conformational change at the beginning of the trajectory, and *d*(S61Oγ-L309O) stayed less than 6 Å throughout the simulation (Figure S10F in the Supporting Information). Second, a stable salt bridge was formed between Neu5Ac and R168 at 100 ns, which persisted until 240 ns. The interaction presented for ~27% of the simulation time (Table S5 in the Supporting Information). It was then replaced by another salt bridge with R57 that lasted until 350 ns. Such a highly stable Neu5Ac-R168 salt bridge, with distance *d*(R168rCζ-C1) maintained at 4 Å for about 70 ns, was not found in any of the other trajectories. Inspection of the dps2 trajectory revealed that, in the absence of noticeable protein conformational changes, movements of local side chains forced by the incoming ligand opened another route in the upper portion of the protein, bypassing the restrictions imposed by TM1 and TM7. This new route permitted Neu5Ac to come very close to the R168 side chain, forming the stable salt bridge. However, the physiological relevance of this route is questionable.

On the other hand, the picture emerging from dps1, such as modest outward-opening of the pore for ligand uptake, can be readily applied to dps3, which only showed some minor variations. The most remarkable difference is the even more extensive Neu5Ac-R57 salt bridge interaction when the ligand traveled in the vicinity of the binding site, which lasted for 60 ns (Figure S12D in the Supporting Information).

In summary, the dp trajectories suggest that the pore outward-opening through protein conformational changes was modest (or nonexistent) when E171 and E175 both stayed deprotonated.

#### 2.2.3. Protonated E171

In p171, E171 is protonated, while E175 stays deprotonated. In sharp contrast with dps1, p171s1 experienced remarkable evolution in protein conformation. Residues with large backbone RMSD were found to be predominantly near the lumen end, including those in TM1, TM5/6, TM7, TM9/10, and TM11/12 (Figure S5B in the Supporting Information).

Inspections of the p171s1 trajectory revealed that the ligand quickly entered the pore at *t* ~ 10 ns, moved down passing L309 at 150 ns into the lower section of the pore, and left the pore at 430 ns. Sialin opened outward for ligand uptake at the beginning of the simulation, and it closed after the ligand departed the upper section of the pore into the binding site. This is evident from *d*(S61Oγ-L309O), which stayed at ~11 Å for 120 ns before gradually dropping to 3–5 Å. On the cytosol side, the pore was initially closed as in the equilibrated p171, then opened when the ligand arrived, and finally closed again after it passed through. The structural changes were captured by *d*(L199Cα-L426Cα), which began a gradual increase from 10 Å at 320 ns to the maximum of 14 Å at 400 ns, followed by a quick descent at 455 ns.

Overall, R57 kept a stable salt bridge with E175, except for a very brief (~1 ns) disruption at *t* ~ 89 ns, during which R57 formed a salt bridge with Neu5Ac. The short-lived salt bridge did not have any noticeable lasting impact on the ligand or protein. Meanwhile, R168 enjoyed larger flexibility (~57% of the time characterized as stable) due to weakened interactions with the protonated E171. The carboxyl group of Neu5Ac interacted modestly with R168 for 30 ns when the ligand traveled in the upper section of the pore, as indicated by *d*(R168Cζ-C1) shortened to 7 Å from 130 to 160 ns. The ligand interacted much more strongly with R57, as *d*(R57Cζ-C1) dropped to below 6 Å, sometimes to as short as 4 Å, from *t* ~ 155 to 320 ns.

Among the representative geometries, Low is similar to 8DWI, Mid deviates from it, and Up delivers the most noticeable differences. This is echoed by the backbone RMSD in column 9 of Table S3: 2.9, 2.2, and 1.8 Å for the Up, Mid, and Low geometries, respectively. The substantial conformational changes in the Up geometry helped the pore open to the lumen, which is confirmed by the HOLE calculations. While the upper section of the path was narrow in the Mid and Low geometries, it is wider near the entrance in the Up geometry. As in equilibrated p171, the lower section of the path in all three geometries featured a bottleneck near L199 and L426, preventing the ligand from freely diffusing through.

The p171s2 and p171s3 trajectories share significant similarity with p171s1. For example, they also witnessed significant conformational changes in many TMs near the lumen end, the closure of the lower-section path to cytosol except when the ligand pushed through, and the occurrence of strong R57-E175 interactions. The three trajectories also exhibited subtle differences. For example, R168 did not interact with Neu5Ac at all in p171s2 or p171s3 (see Figures S14D and S16D in the Supporting Information, respectively). Regarding pore outward opening (Figures S14F and S16F in the Supporting Information), p171s2 underwent modest pore opening on the lumen side, similar to dps1, whereas the outward pore opening in p171s3 was as wide as p171s1, with *d*(S61Oγ-L309O) reaching 11 Å, albeit lasting for a shorter time (~30 ns). Taking all three trajectories into consideration, it seems that protonating E171 may make it easier to achieve pore outward-opening and inward-closing.

#### 2.2.4. Protonated E175

In p175, E171 is deprotonated, and E175 is protonated. Trajectory inspections of p175s1 revealed that the ligand entered the pore at t ~ 30 ns, moved into the lower section of the pore at 210 ns, and left the pore at 440 ns. Like p171s1, p175s1 exhibited protein conformational evolutions, although generally less prominent. Residues with large backbone RMSD were found to be near both the lumen (TM1, TM5/6, and TM11/12) and cytosol (TM4/5) ends (Figure S5C in the Supporting Information). The extent of pore outward-opening was roughly between that of dps1 and p171s1, as characterized by d(S61Oγ-L309O), which reached ~9 Å at t ~ 80 ns, lasted for 100 ns before decreasing, and dropped to 5 Å by 240 ns. On the cytosol side, the pore largely remained partially open, as in the equilibrated p175, until 370 ns, when it widened for the ligand to pass through.

As expected, the R168-E171 salt bridge was very stable (~93% of the time), while the R57-pE175 interaction was perturbed far more frequently (only ~42% of the time characterized as stable). A Neu5Ac-R168 salt bridge was formed briefly between 125 and 145 ns. The ligand interacted more extensively with R57, with d(R168Cζ-C1) < 6 Å from 145 to 170 and from 190 to 290 ns.

Considering the representative geometries when E175 is protonated, not surprisingly, the protein conformations deviated from 8DWI the most in the Up geometry. The backbone RMSD for the Up, Mid, and Low geometries are 2.3, 2.1, and 2.1 Å, respectively (column 13 of Table S3 in the Supporting Information). Overall, the deviations are more substantial than those in dps1 but less significant than those in p171s1. In the Up geometry, the conformational changes assisted the pore in outward opening. The sections of the pore below the binding site were similar in the three geometries. Each geometry possessed at least one bottleneck that prevented the ligand from freely diffusing through.

Qualitatively, p175s2 and p175s3 told the same story as did p175s3. Just like p175s1, the lower section of the path in both p175s2 and p175s3 became wider when the ligand moved through. Also, Neu5Ac interacted with R57 more strongly and more extensively than with R168 in both trajectories. The most remarkable difference from p175s1 is that both the p175s2 and p175s3 trajectories displayed more apparent outward openings (Figures S18F and S20F in the Supporting Information). This is especially true in p175s2, where the opening was almost as large as in p171s1.

To summarize, the behavior of p175 was approximately between that of dp and p171. The pore opening to lumen was generally wider than in dp but not as wide as in p171, while the pore opening to cytosol was narrower than in dp but wider than in p171.

## 3. Discussion

Despite many efforts, a full understanding of the sialin’s operating mechanism is yet to be achieved. The results in this computational study are intended to provide further insights through atomistic-level simulations. It should be emphasized that sialic acids transported by sialin is complicated, and that the trajectories generated in this work only sampled a tiny portion of the entire phase space. Moreover, there are biases and systematic errors in model constructions, including force field parameters, simulation protocols, and so on, which are difficult to gauge. As a non-equilibrium simulation technique, SMD has limitations and may cause artifacts. For example, the gating motions introduced by forced ligand movement may not occur spontaneously in equilibrium simulations, and the observed conformational changes may be induced artifacts of the steering protocol rather than protonation-dependent effects. The pulling speed of 0.1 Å/ns, which is generally considered state-of-the-art and seemed slow enough to enable smooth transports of Neu5Ac through sialin, is still orders of magnitude faster than the migration speed of sialic acid in reality. Thus, the conclusions based on these results must be taken with caution.

### 3.1. Protein Conformational Changes

Starting from the same experimental structure 8DWI, which is inward-facing and partially open, equilibrations ended up with subtly different inward openings in the three models: dp was fully open, p171 became inward-closed, and p175 remained partially open. The closing in p171 was accomplished by bending the cytosol end of TM5, which promoted the hydrophobic interactions between L199 and L426 side chains. The presence of Neu5Ac near the binding site might have hindered large-scale global conformational changes in p171. It is also likely that the protein needs a much longer simulation time, probably orders of magnitude longer, to fully transform the overall global conformation. Nevertheless, the observed trend is in line with the hypothesis that protonation of E171 encourages inward-closing, while deprotonation of both E171 and E175 advocates for inward-opening.

The protein conformational changes along with sialic acid transport were explored by the SMD simulations. The dp model underwent only small conformational changes in sialin, which opened modestly or not at all to the lumen. In contrast, p171 saw more remarkable variations in the protein structure, with large outward openings. The p175 model also experienced decent outward-opening, although on average being less significant than in p171. The extent of protein conformational changes is reflected in the accumulated pulling work curves for the SMD trajectories (Figure S23; the pulling forces are shown in Figure S22, and the corresponding statistical analysis is provided in Table S6, all in the Supporting Information).

As expected, substantial variations were observed in the pulling work curves among the three trajectories for each of the dp, p171, and p175 models. Nevertheless, the average accumulated work is smallest for dp and largest for p171, consistent with the extent of protein conformational changes, which are minimal for dp and most pronounced for p171. Notably, substantial differences in the shapes of the pulling work curves emerge for *t* > 300 ns, when the ligand is located in the second (lower) half of the pore. In the dp model, equilibration results in a widely open pore toward the cytosol, allowing the ligand to move almost freely into the cytosol, with only minor, if any, increases in the accumulated work. In contrast, equilibration in p171 leads to a closed pore, requiring substantial work for Neu5Ac to pass through, as reflected by the most pronounced continuous increase in the accumulated work curve.

Given that the accumulated work values (75–140 kcal/mol) are very large, the barrier for Neu5Ac transport would likely be prohibitively high without changes in the protonation states of E171 and E175.

The above results motivate us to hypothesize that the protonation of E171 or E175 is essential for sialin to undergo conformational change for pore outward-opening (and inward-closing) for ligand uptake. In that regard, the protonation of E171 is probably more impactful. Potential-of-mean-forces calculations will be needed to quantify the free energy barriers associated with the ligand transport under these different conditions to verify this hypothesis.

### 3.2. Representative Geometries

Because the three independent SMD runs of each model visited different regions of the vast phase space, the representative geometries extracted from different trajectories may vary from each other considerably. For example, taking the p171s1 Up geometry as reference, the average backbone RMSD is 2.1 and 2.6 Å for the p171s2 and p171s3 Up geometries, respectively, even though they are all outward-facing. Thus, it is not particularly helpful to compute the RSMD via direct comparisons between the Up (or Mid or Low) geometries across different trajectories. Instead, one can be better informed by tracking how the RMSD evolves from the Up to Mid and finally to Low geometries of a given trajectory against a common reference structure.

As shown in Table S3 in the Supporting Information, the backbone RMSD against 8DWI generally varies with qualitatively similar trends for the trajectories of a given model: the largest for the Up geometry and the smallest for the Low geometry, which is confirmed by the mean RMSD over the s1, s2, and s3. The variations are the smallest in dp, with the mean RMSD decreased by 0.15 Å from 2.13 Å for Up to 1.98 Å for Low. The largest variations are found for p171, with a drop of 1.00 Å from 2.92 to 1.92 Å. The p175 model is in between, with a 0.66 Å difference between 2.76 (Up) and 2.10 (Low) Å. These results exemplify the protein conformational changes that accompany Neu5Ac transport.

### 3.3. R168

The experimental structure of mutant R168K (PDB 8U3F [13]) reveals that residue 168 loses its interactions with the R57 backbone carbonyl group while forming a salt bridge with E171. This locks sialin in the outward-opening conformation, disrupting the sialic acid transport cycle, and may prompt one to conclude that R168 is critical to sialin operation. Our simulations here show that overall, Neu5Ac interacted strongly and extensively with R57 while much less so with R168 (Table S5 in the Supporting Information). Moreover, unlike *d*(S61Oγ-L309O), which clearly signaled the outward pore opening by conformational changes, *d*(R168Cζ-R57O) did not correlate obviously with the outward opening, undermining *d*(R168Cζ-R57O) as an effective metric in measuring the pore opening to the lumen. These results led us to speculate that R168 is not essential to sialin’s operation. What really matters is the protonation and deprotonation of E171, not the presence of R168. Our proposal is actually consistent with the experimental observations that, while mutants R57A [11] and R57K [13] become seriously dysfunctional, R168A retains most of the ligand transport function [11].

Why is mutation R168K impeding sialin operation? Interestingly, our recent quantum model calculations of the nitrate/nitrite antiporter NarK revealed that the arginine-to-lysine mutation of a pore arginine residue near the protein center leads to competition for a proton between the lysine and nitrite, which traps the protein in an intermediate state of the cycle, blocking the ligand transport [16]. This is because a titratable functional group buried deep in a relatively hydrophobic environment tends to see its p*K*a shift toward seven. With p*K*a ~10.5, lysine is more vulnerable than arginine (p*K*a ~12.5) to such impacts, and glutamate (p*K*a ~4.5) is likely affected, too. Given that the salt bridge E171-R168 side chains are also located deep in the sialin interior, we suspect that the R168K mutation causes similar proton sharing between the lysine and E171, effectively keeping E171 in the protonated state.

**Figure 6:**
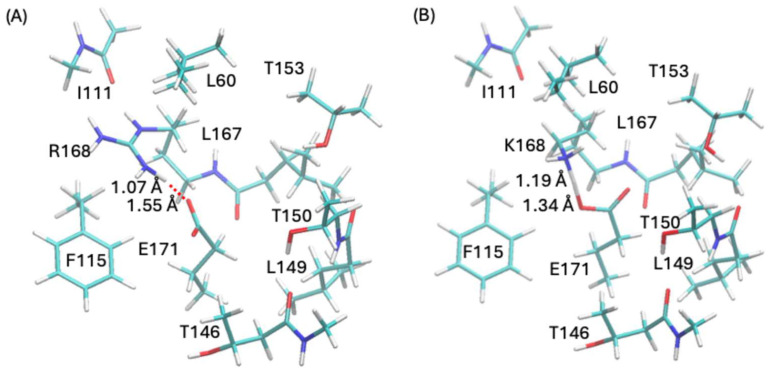
Optimized geometries of (**A**) R168-E171 and (**B**) K168-E171 salt bridges by quantum calculations. Distances d(N-H) and d(H-O) are marked, where N and O are the respective heavy atoms from the residues 168 and 171 that are closest to each other, and H is the hydrogen atom in between. Color code: C, cyan; N, blue; O, red; H, white.

This compelled us to perform additional quantum calculations on two respective truncated models of the R168-E171 and K168-E171 salt bridges surrounded by the nearby residues. The models were constructed, respectively, based on the experimental structure PDB 8U3F [13] (R168K mutant) and 9AYB [13] (S61A mutant), both of which are in the outward-opening conformations. The salt bridges are embraced by residues L60, I111, S114, F115, T146, L149, T150, L164, T153, I165, L167, A169, and L170. The polarizable continuum solvation (PCM) [17] model was employed with 1-butanol (*ε* = 17.332) as the solvent to mimic the local protein environment that is rich in threonine and serine. The density function theory model B3LYP [18,19,20] and the Pople basis set 6-31G [21,22,23,24] were used; such a combination has been shown to yield reasonably accurate energies and geometries with affordable computational expenses in our previous studies of anion channels [16,25,26,27,28]. The D3 empirical dispersion corrections [29] were also included. The calculations were carried out employing the *Gaussian16* package [30]. See Section S5 in the Supporting Information for more computational details. Geometry optimizations revealed that, despite the highly similar initial geometries, in the optimized final geometries, K168 shared its proton with E171 while R168 essentially retained its proton (Figure 6). The results suggest that E171 is effectively kept in the protonated state in R168K. According to our hypothesis, sialin will likely be locked in an outward-opening/inward-closed state, just as what the experimental structure 8U3F unveiled.

Table S7 in the Supporting Information lists the QM atomic charges for the E171 and R/K168 side chain atoms in both the initial and optimized geometries. The changes in atomic charges upon optimization indicate that, as the proton moves toward E171, E171 becomes less negatively charged while R/K168 becomes less positively charged. This effect is more pronounced for K168 than for R168; specifically, in the E171–K168 system, the E171 carboxyl group and Cδ atom gain +0.25 e and +0.20 e, respectively, consistent with a more substantial proton transfer in K168 compared to R168.

Of course, we note that the speculation of the proton sharing was based upon geometry optimizations, which only sampled a small region in the phase space. A more convincing demonstration will be MD simulations that more extensively sample the proton dynamics in this region. To this end, combined QM/MM [31,32,33,34] simulations with the above truncated-QM model as the QM subsystem will be an attractive choice to use and will be pursued in the near future.

### 3.4. Sialic Acid Transport

With the findings in this work, a refined model of sialic acid transport with enriched atomistic details emerges (Figure S9 in the Supporting Information). The transport cycle starts when a proton from the lumen enters the small tunnel in the N-domain and protonates E171, triggering sialin to change conformation from outward-closed/inward-opening to outward-opening/inward-closed, ready for sialic acid uptake. Once the ligand approaches the binding site and forms a salt bridge with R57, the electrostatic changes in the local environment may shift up the p*K*a of E175, prompting it to accept a proton from the protonated E171. With E175 protonated, the pore exit to the cytosol now becomes partially open. The location of E175 allows its side chain to access the well-hydrated lower section of the pore, facilitating proton release via water wires connecting to the cytosol. The proton release may even use the titratable carboxyl group of the sialic acid as part of the proton wire. After the proton departs from E175, with both E171 and E175 deprotonated, sialin fully opens the pore to cytosol, and sialic acid exits. Back to the outward-closed/inward-opening state, sialin is ready for the next cycle.

Although the refined model suggests that E171 protonation occurs before sialic acid uptake, we do not rule out the possibility that the ligand uptake occurs at about the same time as the protonation of E171. However, we suspect that this is less likely. Given the location of E171 deep in the protein center, the electrostatic changes in the local environment caused by the arrival of the ligand at the luminal entrance of the pore are probably not significant enough to really influence the p*K*a of E171. On the other hand, a sialic acid near the binding site is a short distance from E175, and the impact of its negative charge on the p*K*a of E175 can be more substantial. How close is close enough? We do not know, and further investigations need to figure this out.

## 4. Methods

### 4.1. Model Constructions and Equilibrations

Beginning with the WT protein’s cryo-EM structure by Zheng and coworkers (PDB 8DWI [11]), which will be denoted “8DWI” in this paper, we docked the ligand Neu5Ac along the sialic acid translocation path using the Autodock Vina [35,36] program. In total, 90 trials were performed with different random seeds, and the results converge reasonably well. In most poses of high docking scores, the carboxyl group of the Neu5Ac interacts with the positively charged R57 side chain. The pose with the highest docking score was selected for the subsequent model constructions. See Section S1 in the Supporting Information for more details.

With the docked ligand, three atomistic models of WT sialin, which were named “dp” (stands for deprotonated E171 and E175), “p171” (stands for protonated E171), and “p175” (stands for protonated E175), respectively, were constructed using the CHARM-GUI [37] program. The protonation states of titratable residues were assigned according to the PROPKA [38] analysis (Section S2 in the Supporting Information) except for those of E171 and E175. Both E171 and E175 were deprotonated in dp; only E171 was protonated in p171, and only E175 was protonated in p175. Additional details of E171 and E175 side chain protonation are provided in Section S3 in the Supporting Information. The protein was embedded in a bilayer of 410 POPC lipid molecules, with 205 lipids in the upper and lower leaflets each. In our earlier experiments, sialin has been demonstrated to be functional when embedded in either High-Five cell plasma membrane or soybean polar extract lipid nanodiscs [11]. The composition of the membrane appears to have no significant influence on transporter function. On the other hand, POPC-only lipids had been adopted in many of our previous simulations of transport proteins with satisfactory results [16,27,39,40], and thus we continued to use a POPC-only lipid bilayer in this work. The membrane was placed in the *x*-*y* plane. The sialic acid translocation path was aligned approximately with the *z* axis, with the lumen end in the +*z* direction and cytosol end in the –*z* direction. Each model was solvated by ~41,000 TIP3P [41] water molecules. To achieve charge neutrality and a 0.15 M physiological solution concentration, 111 potassium and, depending on the protonation states of E171 and E175, 114 or 115 chloride ions were included. The CHARMM36m force fields were selected for the protein, lipids, and ions [42,43,44,45,46]. The CHARMM General Force Field (CGenFF) [47] was selected for Neu5Ac (residue name ANE in this work), the structure of which, together with the involved atom names, atom types, and atomic charges, is provided in Table S1 in the Supporting Information. Periodic boundary conditions were applied, and the dimensions of the primary cell containing ~185,000 atoms were ~125 × 125 × 127 Å^3^.

After initial energy minimizations and heating (from 0 to 300 K), each model was equilibrated as an isothermal–isobaric (*NpT*) ensemble at 300 K and 1 bar. All MM calculations in this work were performed using the NAMD (version 2.10) program [48]. A cutoff of 12 Å was used for nonbonded short-range interactions, with a smoothing function switched on at 11 Å. The Particle Mesh Ewald [49] method was employed for long-range electrostatic interactions. A time step size of 2 fs was used, with the RATTLE [50] algorithm invoked to keep the X-H (X=C, N, and O) bonds rigid, and structures along the trajectory were saved every 0.1 ns. Equilibrations were run for 420 ns for dp and p175 and 250 ns for p171. The generated trajectories are denoted “dpeq”, “p171eq”, and “p175eq”, respectively, where “eq” stands for equilibration. An in-house code based on the statistical tests described in Ref. [51] was utilized to check if the models had been properly equilibrated.

We note that a long luminal L2 loop (residues 69 to 101) was missing in the adopted experimental structure [11]. To check if this loop might have affected the equilibrated protein conformation, an additional model was constructed by manually adding the L2 loop in dp and equilibrated for 200 ns. Comparing the trajectories with dp, however, did not find any significant difference in the protein conformations, as both models remain stable in a conformation similar to that of the structure 8DWI. The L2 loop was highly flexible, stayed in the lumen solution, and did not interfere with the sialic acid transport path. Further details about the L2 loop are given in Section S4 in the Supporting Information. As such, it was deemed that the L2 loop is not essential to the present models and could be safely neglected in this work.

### 4.2. SMD Simulations

All SMD calculations were carried out at a constant slow velocity of 0.1 Å/ns with a spring constant of 10 kcal/mol/Å^2^. The ligand’s C6 atom, which is very close to the ligand’s center of mass (COM), was steered; as verified in our simulations, the C6 atom is usually less than 0.3 Å away from and always within 1 Å of the ligand COM, despite the conformational variations in the ligand during SMD. Three protein atoms (Cα atoms of G176, F299, and F405) were restrained to prevent the protein from moving along with the ligand; the very small number of restrained protein atoms, the locations of which are near the *x*-*y* plane where the protein’s center of mass is located, helped minimize impacts of these artificial restraints in the SMD simulations on the protein’s possible conformational changes conjectured based on the proposed mechanism (Figure 2).

For each model, three independent sets of SMD runs started from different frames extracted from the equilibrated trajectory, leading to a total of 9 sets of SMD simulations. It all started with Neu5Ac in the putative binding site. For each set of SMD runs, two separate subsets of SMD simulations were performed using the same assigned initial velocities, where Neu5Ac, from its starting location, was pulled *up* in the +*z* direction toward the lumen and *down* in the –*z* direction toward the cytosol, respectively. The pulling stopped after the ligand crossed the membrane, as judged by comparing with the *z* coordinate of the C6 atom of the Neu5Ac with the average *z* coordinate of the phosphorous atom of the lipids in the upper or lower leaflet. At the end point, the ligand was seen either already departed or about to depart from the protein. (Note that, although the reference point moved strictly at a constant speed, the ligand did so only approximately, and this led to various simulation times in different sets of SMD simulations.) The downward-pulling trajectory was reversed and then joined with the upward-pulling trajectory to generate a complete trajectory of outward translocation for the ligand. The 9 trajectories are named “dps1”, “dps2”, “dps3”, “p171s1”, … “p175s3”, respectively, where “s1”, “s2”, and “s3” denote the 1st, 2nd, and 3rd SMD runs, respectively. A summary of all SMD trajectories is given in Table S2 in the Supporting Information, where the equilibration runs above are also described.

### 4.3. Pore Radius Calculations

Based on the structure 8DWI, the pore is roughly 40 Å long. We divided it into two sections: an upper section of ~12 Å long above the Cα atom of L309 and a lower section below it. The upper section is seen close to ligand transport in the structure 8DWI. The binding site is located in the middle of the protein near the top of the lower section. The pore radii along the ligand translocation path were calculated employing the HOLE suite of tools [52] for representative protein geometries extracted from the saved trajectories. In these extracted snapshots, Neu5Ac was located differently: in the upper section of the path crossing the membrane (denoted “Up”), or in the middle of the protein near the binding site (denoted “Mid”), or in the lower section of the path crossing the membrane (denoted “Low”). The Up and Low geometries correspond approximately to the moments when the ligand entered and exited the pore, respectively. For the equilibration runs, only the Mid geometries were extracted near the end of the equilibrated trajectories, as the ligand remained in or near the binding site during the simulations. More details on the extractions of representative geometries are provided in Table S2 in the Supporting Information. For comparison, we also computed the pore radii for the structure 8DWI. The “hard-core” van der Waals radii [53] were used, which implicitly account for the thermal motions of atoms. The location of the C6 atom in Neu5Ac in each geometry was used to initiate the pore search by the HOLE program, except for the Low geometries of dps2 and p171s2, where the searches were initiated employing the locations of the C6 atom in the corresponding Mid geometries.

## Data Availability

The data that support the findings of this study are available at DOI: 10.5281/zenodo.19805045.

## Supplementary Materials

The following supporting information can be downloaded at: https://www.mdpi.com/article/10.3390/ijms27104629/s1.

## Abbreviations

The following abbreviations are used in this manuscript:

SLC17	Solute carrier 17
SA	Sialic acid
WT	Wild type
TM	Transmembrane
Neu5Ac	*N*-acetylneuraminic acid
RMSD	Root means square deviation
NAAG	*N*-acetylaspartylglutamate
MD	Molecular dynamics
SMD	Steered molecular dynamics
MM	Molecular mechanics
QM	Quantum mechanics
QM/MM	Quantum-mechanics/molecular-mechanics
POPC	1-Palmitoyl-2-oleoyl-*D*-glycero-1-phosphatidylcholine
pE171	Protonated E171
pE175	Protonated E175
COM	Center of mass
