# Supplementary material for: Protonation States of Proton-Sensing Glutamate Residues in Transporter Sialin
Source: Int J Mol Sci. 2026 May 21;27(10):4629. doi: 10.3390/ijms27104629 (PMC13207226; doi:10.3390/ijms27104629)

5/10/2026

Supporting Information for

Protonation States of Proton-Sensing Glutamate Residues in Sialin Transport

Eric Wooten,[1] Nara L. Chon,[1] Muhamadjon Dzhalolov,[1] Hongjin Zheng,[2] and Hai Lin[1,3]**

[1]Department of Chemistry, University of Colorado Denver, Denver, Colorado, 80217 USA

[2]Department of Microbiology, University of Alabama at Birmingham, Birmingham, AL, 35294 USA

[3]Center of Advanced Computational Molecular Sciences, University of Colorado Denver, Denver, Colorado, 80217 USA

*Correspondence: zhengh@uab.edu, hai.lin@ucdenver.edu

Table of Contents

S1. Additional details of docking calculations. 4
S2. Additional details of PROPKA analysis. 4
S3. Additional details of E171 and E175 sidechain protonation. 6
S4. Additional details of the L2 Loop. 8
S5. Additional details of quantum model calculations for the R/K168-E171 salt bridge 9
Table S1. CGenFF force fields for the ligand Neu5Ac. 10
Table S2. Summary of MD runs. 11
Table S3. Backbone RMSD (Å) of representative geometries. [a] 12
Table S4. Stability of salt bridges in simulations. [a] 13
Table S5. Interactions between Neu5AC carboxyl group and R57/R168 in simulations. [a] 14
Table S6. Summary of pulling forces and accumulated works in SMD simulations 15
Table S7. QM atomic charges for sidechain functional groups in R/K168-E171 salt bridge. [a] ... 16
Figure S1. Backbone RMSD of individual residues averaged over time in equilibration. 17
Figure S2. Overlays of equilibrated geometries and experimental structure. 18
Figure S3. Pores of equilibrated geometries and experimental structure. 19
Figure S4. Ligand atom *z* coordinates over simulation time during equilibrations. 20
Figure S5. Backbone RMSD of individual residues averaged over time in SMD s1 trajectories. 21
Figure S6. Ligand atom *z* coordinates over simulation time in SMD s1 trajectories. 22
Figure S7. Overlays of representative geometries in SMD s1 trajectories and experimental structure 23
Figure S8. Pores of representative geometries in SMD s1 trajectories and experimental structure. 24
Figure S9. Sialic acid transport model with enriched atomistic details. 25
Figure S10. Additional plots for trajectory dps2. 26
Figure S11. Pores of representative geometries in trajectory dps2. 27
Figure S12. Additional plots for trajectory dps3. 28
Figure S13. Pores of representative geometries in trajectory dps3. 29
Figure S14. Additional plots for trajectory p171s2. 30
Figure S15. Pores of representative geometries in trajectory p171s2. 31
Figure S16. Additional plots for trajectory p171s3. 32
Figure S17. Pores of representative geometries in trajectory p171s3. 33
Figure S18. Additional plots for trajectory p175s2. 34
Figure S19. Pores of representative geometries in trajectory p175s2. 35

Figure S20. Additional plots for trajectory p175s3. 36
Figure S21. Pores of representative geometries in trajectory p175s3.. 37
Figure S22. Pulling force against time in SMD simulations. 38
Figure S23. Accumulated pulling work against time in SMD simulations. 39

S1. Additional details of docking calculations.

Beginning with the WT protein's cryo-EM structure by Zheng and coworkers (PDB 8DWI), the ligand Neu5Ac was docked along the sialic acid translocation path using the Autodock Vina program. In total, 90 trials were performed with different random seeds. The dimension of the binding-site box center was 16, 18, and 20 Å in the x, y, and z directions, respectively, with the box center located at (123.2, 132.9, 118.0) Å. The grid-point spacing was 1 Å in all directions. The following residues are included: F50, Y54, A55, R57, V58, N59, S61, Y119, Q123, E175, G176, F179, S0202, Y203, A204, G205, A206, Q207, L208, G209, T210, V211, I212, S213, L214, H298, F299, Y301, N302, T304, F305, Y306, L308, L309, L312, S332, L333, P334, Y335, L336, G337, S338, W339, M342, T400, G404, C406, S407, S308, F410, and S411. The results converge reasonably well. In most poses of high docking scores, the carboxyl group of the Neu5Ac interacts with the positively charged R57 sidechain. The pose with the highest docking score was selected for the subsequent model constructions.

S2. Additional details of PROPKA analysis.

The protonation states of titratable residues were assigned according to the PROPKA analysis except for those of E171 and E175, which are manually assigned in the dp, p171, and p175 models. The pH was set to 7.4. The resulting p*K*a of the titratable residues are given as follows:

- ASP (intrinsic p*K*a = 3.80):

Residue	D66	D104	D158	D250	D350	D388	D416	D450
p*K*a	5.15	3.44	4.31	4.50	4.09	4.06	3.34	5.00

  Accordingly, all aseptate residues were deprotonated.

- GLU (intrinsic p*K*a = 4.50):

Residue	E106	E171	E175	E194	E262	E264	E318	E326	E455	E481
p*K*a	4.24	6.86	5.94	3.79	6.08	4.15	6.34	4.80	3.47	4.57

  Accordingly, all glutamate residues were deprotonated except E171 and E175 as noted above.

- HIS (intrinsic p*K*a = 6.50):

Residue	H183	H255	H260	H298	H414

p*K*a	4.80	5.23	6.40	3.91	4.48

Accordingly, all histidine residues were charge neutral with protonation at the δ positions.

- CYS (intrinsic p*K*a = 9.00):

Residue	C35	C36	C221	C341	C362	C387	C406
p*K*a	9.43	9.16	13.51	10.69	9.13	9.34	12.49

Accordingly, all cystine residues stayed charge neutral.

- TYP (intrinsic p*K*a = 10.00):

Residue	Y40	Y54	Y117	Y119	Y128	Y203	Y222	Y223	Y228	Y231
p*K*a	10.17	10.89	10.88	15.06	10.11	12.00	10.19	10.53	9.58	10.50
Residue	Y261	Y265	Y301	Y306	Y315	Y335	Y389	Y421	Y461	
p*K*a	10.05	11.45	13.52	12.52	12.18	13.86	10.41	10.10	10.23	

Accordingly, all cystine residues stayed charge neutral.

- LYS (intrinsic p*K*a = 10.50):

Residue	K132	K136	K197	K254	K256	K263	K278	K287	K317
p*K*a	10.43	9.96	11.85	10.43	10.41	10.39	10.26	10.39	8.43
Residue	K355	K445	K479						
p*K*a	10.37	8.61	10.44						

Accordingly, all lysine residues were protonated.

- ARG (intrinsic p*K*a = 12.50):

Residue	R39	R57	R168	R195	R257	R271	R321	R353	R364	R365
p*K*a	13.26	16.39	10.84	11.77	12.32	12.36	12.45	12.24	11.25	12.10

Accordingly, all arginine residues were protonated.

S3. Additional details of E171 and E175 sidechain protonation.

In this work, we constructed and equilibrated three models: (i) **dp**, in which both E171 and E175 are deprotonated; (ii) **p171**, in which E171 is protonated and E175 remains deprotonated; and (iii) **p175**, in which E175 is protonated and E171 remains deprotonated.

We did not simulate a model in which both E171 and E175 are simultaneously protonated. Although we cannot completely rule out the possibility of such double protonation, we consider it to be of very low probability. The proposed transport cycle, which is based on experimental structures and biochemical measurements, does not indicate simultaneous protonation of both E171 and E175.

If E171 and E175 were both protonated during a transport cycle, maintaining the established 1:1 stoichiometry of sialic acid/proton cotransport would require either (i) the transport of two sialic acid molecules together with two protons within a single cycle, which is unlikely, or (ii) that E171 and E175 will not deprotonated concurrently, which does not align very well with the PROPKA analysis of the experimental structure 8DWI (**Section S2**).

In the experimental structure 8DWI, OE1 of E171 is closer to the R168 side chain than OE2, with the shortest O–N distances of 3.4 and 5.4 Å, respectively. In the p171 model, the proton was added to OE2; therefore, this protonation scheme is expected to minimally disrupt the E171–R168 salt bridge.

On the other hand, in the 8DWI structure, OE2 of E175 is closer to the R57 side chain than OE1, with the shortest O–N distances of 3.0 and 4.7 Å, respectively. Although the proton was also added to OE2 in the p175 model, during equilibration the carboxylate group quickly (within ~0.5 ns) rotated such that OE1 became oriented toward the R57 side chain, with an O–N distance of 2.2 Å. This geometry is well suited for maintaining the E175–R57 salt bridge.

During equilibration, the protonated carboxyl groups rotated frequently in both the p171 and p175 models. Specifically, the dihedral angle φ(OE1–CD–CG–CB) ranged from −151° to 159° for protonated E171 (pE171) in the p171 model and from −164° to 166° for protonated E175 (pE175) in the p175 model, indicating that both rotamers were sampled, as shown in the plots below.

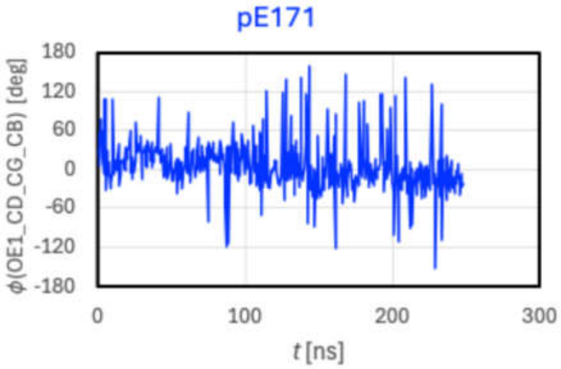

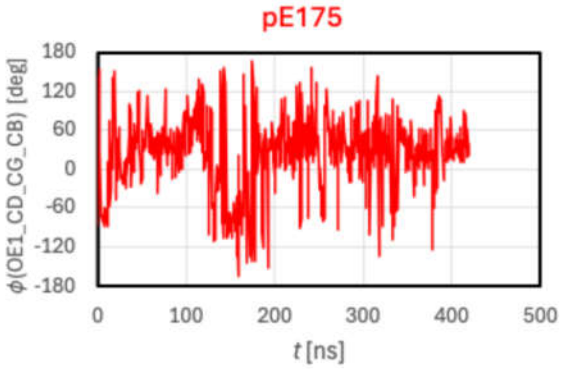

Taken together, these observations suggest that the initial choice of proton placement (OE1 versus OE2) in p171 or p175 does not significantly affect the structural integrity of the relevant salt bridges or the overall simulation results.

S4. Additional details of the L2 Loop.

In the literature, no specific studies have directly examined how the L2 loop (residues D69 to Y101) affects Sialin transport function. From the published high-resolution structures (Hu, et al. Sci. Adv. 2023, 9, eade8346; Schmiege, et al. *Nat. Commun.* 2024, 15, 4386), it is clear that the L2 loop is highly flexible and does not appear to either influence the conformational changes required for transport or the substrate specificity. Therefore, we hypothesize that the L2 loop plays no direct role in Sialin-mediated transport. To experimentally test this, Sialin mutants with the L2 loop deleted or replaced by a shorter loop will need to be generated, expressed in cells, and evaluated using transport assays; however, such experiments will be technically challenging and time-consuming, and they are not conducted in this work.

The glycocalyx on the luminal side of the lysosomal membrane is primarily composed of the abundant structural proteins LAMP-1 and LAMP-2 (Wilke, et al. *BMC Biol.*, 2012, 10, 62.). However, there is no evidence of direct physical interactions between Sialin and LAMP proteins. The L2 loop contains three aspartate residues that can undergo N-glycosylation. Mutagenesis studies have shown that this glycosylation is not essential for transport function (Hu, et al. Sci. Adv. 2023, 9, eade8346). Thus, the flexible, glycosylated L2 loop may help protect Sialin from proteolytic degradation by lytic enzymes in the lysosome. Nevertheless, whether the L2 loop directly interacts with the glycocalyx remains unknown.

In our simulations of the additional model (both E171 and E175 stayed deprotonated) where the L2 loop had been manually added, the L2 loop was highly flexible, stayed in the lumen solution, and did not interfere with the sialic acid transport path. For illustration propose, three representative snapshots at 1 ns (left), 100 ns (middle), and 200 ns (right) are shown below, respectively, where the protein is displayed as helices and coils, and the L2 loop is colored in red.

1 ns 100 ns 200 ns

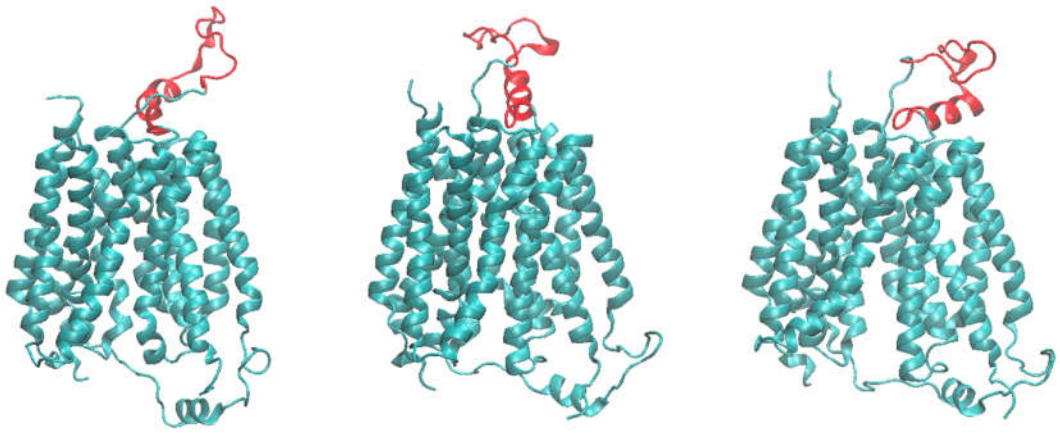

S5. Additional details of quantum model calculations for the R/K168-E171 salt bridge.

Two truncated models of the R/K168-E171 salt bridges were constructed based on the experimental structures. Specifically, Model R168-E171 was built based on PDB 9AYB of mutant S61A, and Model K168-E171 on PDB 8U3F of mutant R168K, both of which are found in the outward-opening conformations. In both 9YAB and 8U3F, the positions and orientations of the residues within 5 Å of the salt bridges are almost identical.

Apart from the sidechain (sc, including the Cα atom) of R/K168 and E171, each models includes the following residues: L60 (sc only), I111 (backbone, or bb, only), S114, F115 (sc only), T146, L149, T150, T153 (sc only), and L167. The backbones are truncated in such a way that they are terminated at Cα atoms. For example, For I111, we include the Cα atoms of both residues 111 and 112. In total, Model R168-E171 contains 163 atoms, and K168-E171 has 161 atoms. The polarizable continuum solvation (PCM) model was employed with 1-butanol (ε = 17.332) as the solvent to mimic the local protein environment that is rich in threonine and serine.

Geometry optimizations were carried out employing the *Gaussian16* package. The density function theory model B3LYP and the 6-31G basis set were used, with the D3 empirical dispersion corrections included. The heavy atoms of residues surrounding the residues 168 and 171 were fixed at their (experimental) positions, as so were the Cα atoms of residues 168 and 171. The positions of the other atoms are fully optimized. The initial geometries are displayed below for Models R168-E171 (left) and K168-E171 (right), with the distances d(N-H) and d(H-O) marked, where N and O are the respective heavy atoms from the residues 168 and 171 that are closest to each other, and H is the hydrogen atom in between:

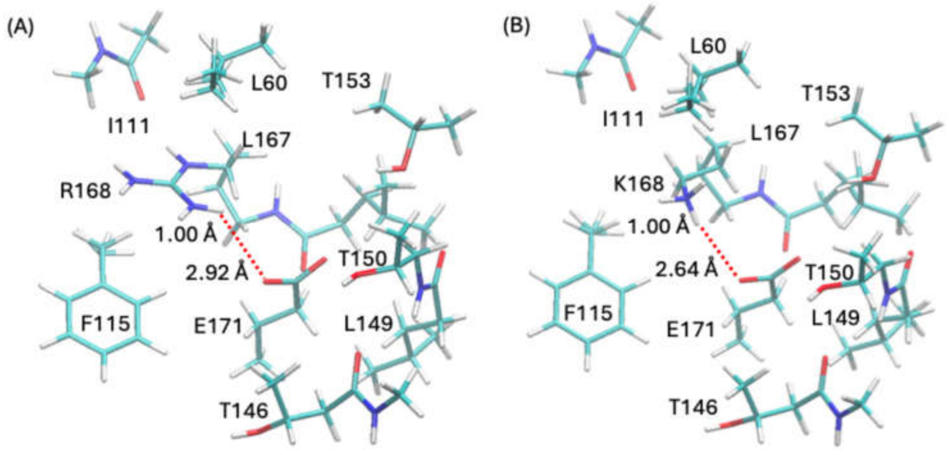

Table S1. CGenFF force fields for the ligand Neu5Ac.

Structure (Residue name as ANE5), Atom Names, Atom Types, and Atomic Charges (e)

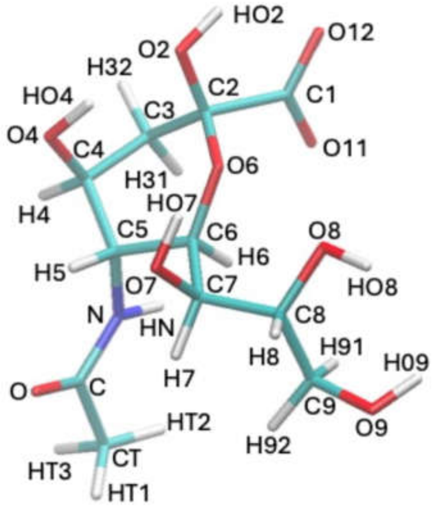

C1	CC2O2	0.30
O11	OC2D2	-0.60
O12	OC2D2	-0.60
C2	CC3062	0.33
O2	OC311	-0.65
HO2	HCP1	0.42
C6	CC3163	0.11
H6	HCA1	0.09
O6	OC3C61	-0.40
C3	CC3261	-0.18
H31	HCA2	0.09
H32	HCA2	0.09
C4	CC3161	0.14
H4	HCA1	0.09
O4	OC311	-0.65
HO4	HCP1	0.42
C5	CC3161	0.07
H5	HCA1	0.09
N	NC2D1	-0.47
HN	HCP1	0.31
C	CC2O1	0.51
O	OC2D1	-0.51
CT	CC331	-0.27
HT1	HCA3	0.09
HT2	HCA3	0.09
HT3	HCA3	0.09
C7	CC312	0.14
H7	HCA1	0.09
O7	OC311	-0.65
HO7	HCP1	0.42
C8	CC312	0.14
H8	HCA1	0.09
O8	OC311	-0.65
HO8	HCP1	0.42
C9	CC322	0.05
H91	HCA2	0.09
H92	HCA2	0.09
O9	OC311	-0.65
HO9	HCP1	0.42

Table S2. Summary of MD runs.

Run	Trajectory	Model	$q(171)/q(175)$ [e]	Run Time [ns]	Time of Extracted Geometries [a] [ns]		
					Up	Mid	Low
Equilibration							
1	dpeq	dp	–1/–1	420	n/a	400	n/a
2	p171eq	p171	0/–1	250	n/a	240	n/a
3	p175eq	p175	–1/0	420	n/a	400	n/a
SMD							
4	dps1	dp	–1/–1	530	30	240	460
5	dps2	dp	–1/–1	504	10	250	445
6	dps3	dp	–1/–1	425	0	200	390
7	p171s1	p171	0/–1	470	10	210	430
8	p171s2	p171	0/–1	435	10	210	410
9	p171s3	p171	0/–1	416	10	210	405
10	p175s1	p175	–1/0	500	30	210	440
11	p175s2	p175	–1/0	507	90	280	460
12	p175s3	p175	–1/0	500	10	210	420

[a.] Up, Mid, and Low denote, respectively, the position of the ligand in the upper sections of the path crossing the membrane, in the middle section of membrane near the binding site, and in the lower sections of the path crossing the membrane.

Table S3. Backbone RMSD (Å) of representative geometries. [a]

	Equilibration			SMD											
Model	dp	p171	p175	dp				p171				p175			
Traj.	eq	eq	eq	s1	s2	s3	Mean	s1	s2	s3	Mean	s1	s2	s3	Mean
Up	n/a	n/a	n/a	2.02	2.14	2.23	2.13	2.91	2.52	3.34	2.92	2.34	3.11	2.83	2.76
Mid	1.52	1.94	1.81	1.78	2.10	2.22	2.03	2.17	2.30	2.34	2.27	2.12	2.56	2.07	2.25
Low	n/a	n/a	n/a	1.91	2.03	2.00	1.98	1.79	2.08	1.90	1.92	2.06	1.89	2.36	2.10

[a] With respect to the experimental structure 8DWI and average over residues A38-L247 and W282-N488.

Table S4. Stability of salt bridges in simulations. [a]

Traj.	R57Cζ_E175Cδ			R168Cζ_E171Cδ		
	Stable	Unstable	broken	Stable	Unstable	broken
**Equilibration**						
dpeq	99.9	0.1	0.0	99.3	0.7	0.0
p171eq	100.0	0	0.0	64.7	35.1	0.2
p175eq	20.4	53.9	25.7	99.3	0.7	0.0
**SMD**						
dps1	86.8	12.6	0.6	94.5	5.3	0.2
dps2	100.0	0	0.0	87.4	10.9	1.7
dps3	94.6	5.2	0.2	98.4	1.6	0.0
p171s1	99.6	0.2	0.2	56.7	42.1	1.2
p171s2	96.6	3.1	0.3	72.5	27.5	0.0
p171s3	99.2	0.8	0.0	81.0	19	0.0
p175s1	41.7	57.9	0.4	93.0	7	0.0
p175s2	28.8	29.8	41.4	97.7	2.3	0.0
p175s3	50.4	48.5	1.1	95.6	4.4	0.0

[a] Shown as percentage in saved frames for each trajectory. Salt bridge distances measured between the heavy atoms closest to the charge center of the involved functional groups, e.g., the Cζ atom of arginine, the Cδ atom of glutamate, and the Nζ atom of lysine. A salt bridge is viewed as stable if the distance remains <5.5 Å for the Arg-Glu and <5.0 Å for Glu-Lys pairs, completely broken if the distance is >8.0 Å for the Arg-Glu and >7.5 Å for Glu-Lys pairs, and unstable in between.

Table S5. Interactions between Neu5Ac carboxyl group and R57/R168 in simulations. [a]

Traj.	R57Cζ_ANEC1			R168Cζ_ANEC1		
	Present (Stable)	Present (Unstable)	Absent	Present (Stable)	Present (Unstable)	Absent
**Equilibration**						
dpeq	89.6	9.9	0.5	0.0	0.0	100.0
p171eq	93.5	6.1	0.4	0.0	0.0	100.0
p175eq	78.9	17.1	4.0	0.0	0.0	100.0
**SMD**						
dps1	0.1	9.2	90.7	0.0	0.0	100.0
dps2	19.6	6.8	73.6	27.4	3.8	68.8
dps3	23.5	16.6	59.9	0.0	0.0	100.0
p171s1	25.2	12.2	62.6	0.0	7.6	92.4
p171s2	17.2	8.7	74.1	0.0	0.0	100.0
p171s3	19.1	19.7	61.2	0.0	0.0	100.0
p175s1	10.2	23.1	66.7	4.5	10.6	84.9
p175s2	27.7	24.3	48.0	0.1	16.1	83.8
p175s3	18.7	20.4	60.9	3.3	21.5	75.2

[a] Shown as percentage in saved frames for each trajectory. Based on distances measured between the heavy atoms closest to the charge center of the involved functional groups, i.e., the carboxyl carbon of Neu5AC and the Cζ atom of an arginine residue. The interaction is considered present and stable if the distance is <5.5 Å, present but unstable if the distance is between 5.5 and 8.0 Å, and absent if the distance is >8.0 Å.

Table S6. Summary of pulling forces and accumulated works in SMD simulations. [a]

	dps1	dps2	dps3	p171s1	p171s2	p171s3	p175s1	p175s2	p175s3
$F_{z,\max}$	924	602	561	693	640	483	553	1282	635
$-F_{z,\max}$	948	936	975	1202	1121	1020	862	898	1504
W	80.5	53.0	92.9	153.3	131.8	132.1	83.1	96.3	122.0
$\langle W \rangle$		75.5			139.1			100.5	
σ_W		16.7			10.1			16.2	

[a] $F_{z,\max}$ and $-F_{z,\max}$ are the maximum forces (in pN) along the +z and -z directions, respectively. The accumulated work W (in kcal/mol) is taken at the end of the given simulation. The mean $\langle W \rangle$ and standard deviation σ_W are given for each set of SMD simulations.

Table S7. QM atomic charges for sidechain functional groups in R/K168-E171 salt bridge. [a]

R168-E171

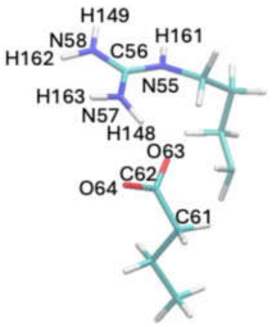

K168-E171

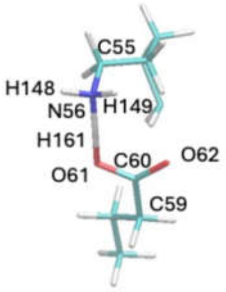

Atom	q_{init}	q_{oph}	$\Delta q = q_{init} - q_{opt}$	Atom	q_{init}	q_{opt}	$\Delta q = q_{init} - q_{opt}$
E171				E171			
C61	−0.196	−0.170	0.026	C59	−0.195	−0.161	0.033
C62	0.165	0.195	0.030	C60	0.170	0.216	0.046
O63	−0.525	−0.423	0.103	O61	−0.519	−0.428	0.091
O64	−0.521	−0.481	0.041	O62	−0.514	−0.432	0.082
R168				K168			
N55	−0.439	−0.433	0.007	C55	−0.035	−0.044	−0.009
H161	0.344	0.315	−0.029	N56	−0.578	−0.581	−0.003
C56	0.427	0.402	−0.025	H148	0.399	0.348	−0.052
N57	−0.566	-0.577	−0.012	H149	0.387	0.338	−0.049
H148	0.349	0.295	−0.054	H161	0.372	0.296	−0.077
H163	0.373	0.341	−0.032				
N58	−0.572	−0.587	−0.015				
H149	0.360	0.330	−0.030				
H162	0.371	0.354	−0.017				

[a] CM5 charges (in e), where q_{init} and q_{opt} are the charge associated with the initial and optimized geometries, respectively. The sidechains of R/K168 and E171 are shown as sticks (color code: C, cyan; H, white; O, red, and N, blue).

Figure S1. Backbone RMSD of individual residues averaged over time in equilibration.

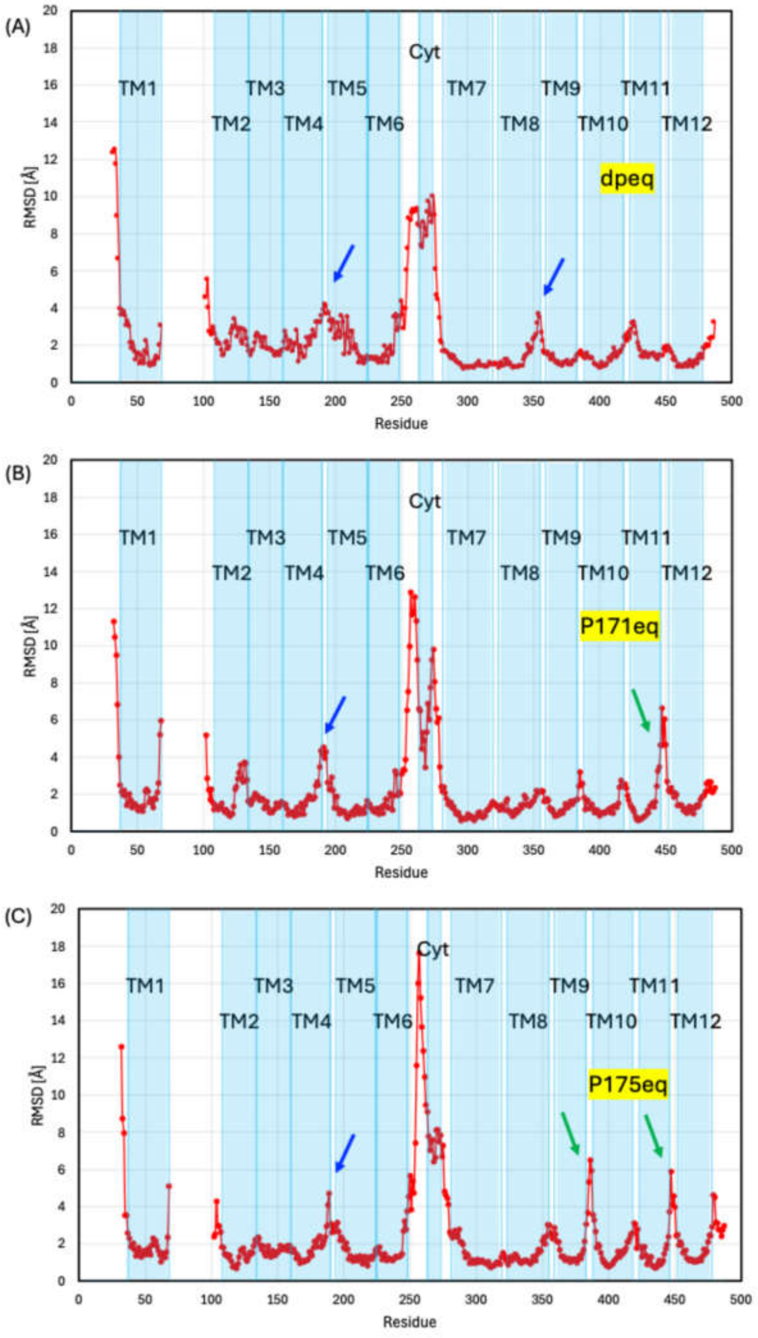

For models (**A**) dp, (**B**) p171, and (**C**) p175. The gap between TM1 and TM2 is due to the missing lumen loop in the experimental structure (PDB 8DWI). Cyt denotes the cytosol helix. Residues in TM and inter-TM loops (excluding the N-terminal and cytosol loops) with large RMSD (> 4 Å) are indicated by blue/green arrows if located on the cytosol/lumen side.

Figure S2. Overlays of equilibrated geometries and experimental structure.

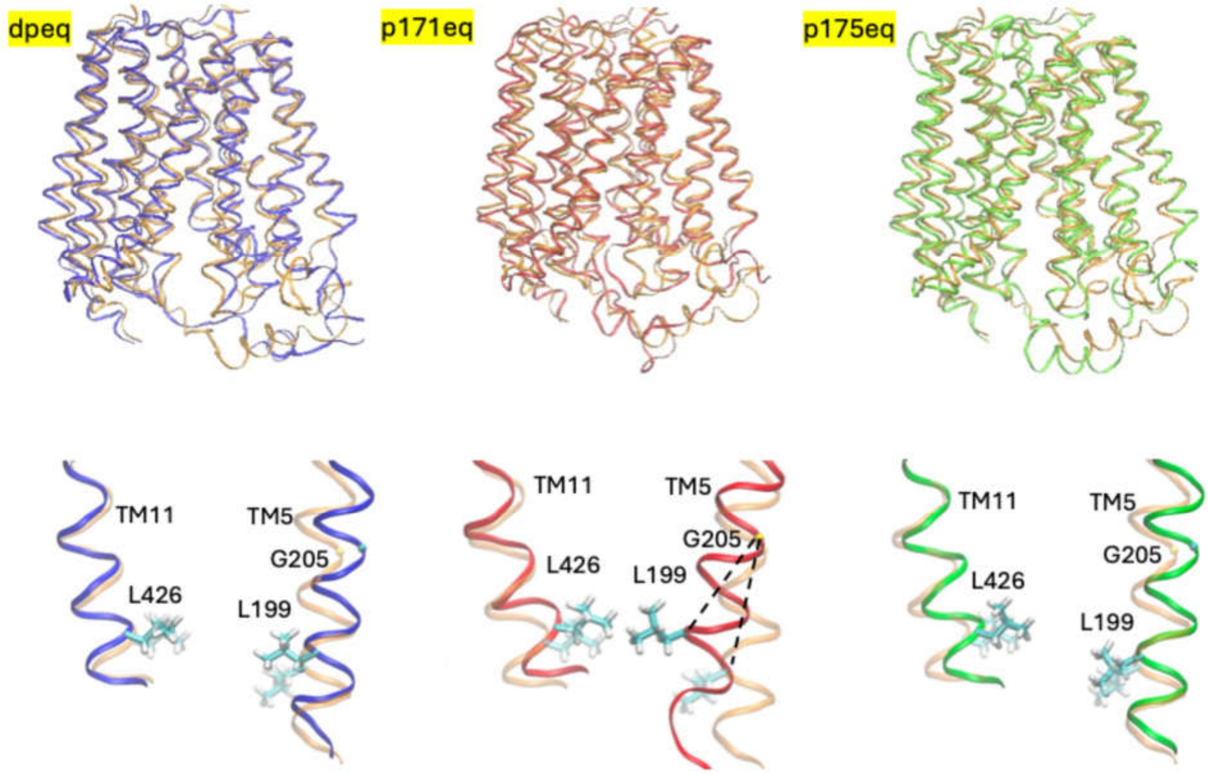

All proteins are shown as helixes and loops. The experimental structure (PDB 8DWI) is in orange color. The representative equilibrated geometries are in blue for dp, red for p171, and green for p175, respectively. The lower panels compare L199-L426 interactions in different models, where the two residues are displayed as sticks (C, cyan; H white), opaque in equilibrated geometries while transparent in 8DWI.

Figure S3. Pores of equilibrated geometries and experimental structure.

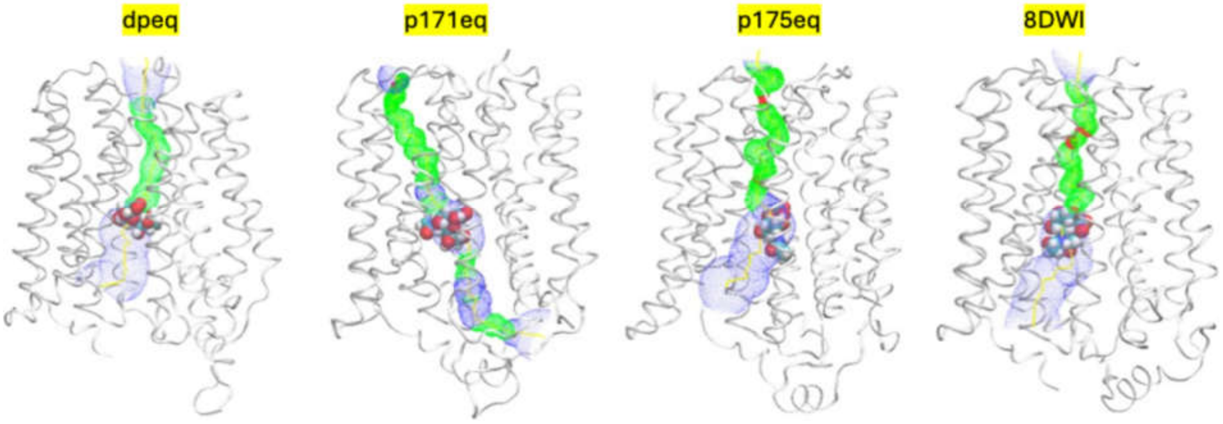

For the pore, red color indicates narrow sections (too tight for one water molecule), blue color wide sections (can accommodate two water molecules), and green color sections of medium-widths. The protein is displayed as helixes and loops in gray, and the ligand as spheres (color code: C, cyan; H, white; O, red, and N, blue).

Figure S4. Ligand atom *z* coordinates over simulation time during equilibrations.

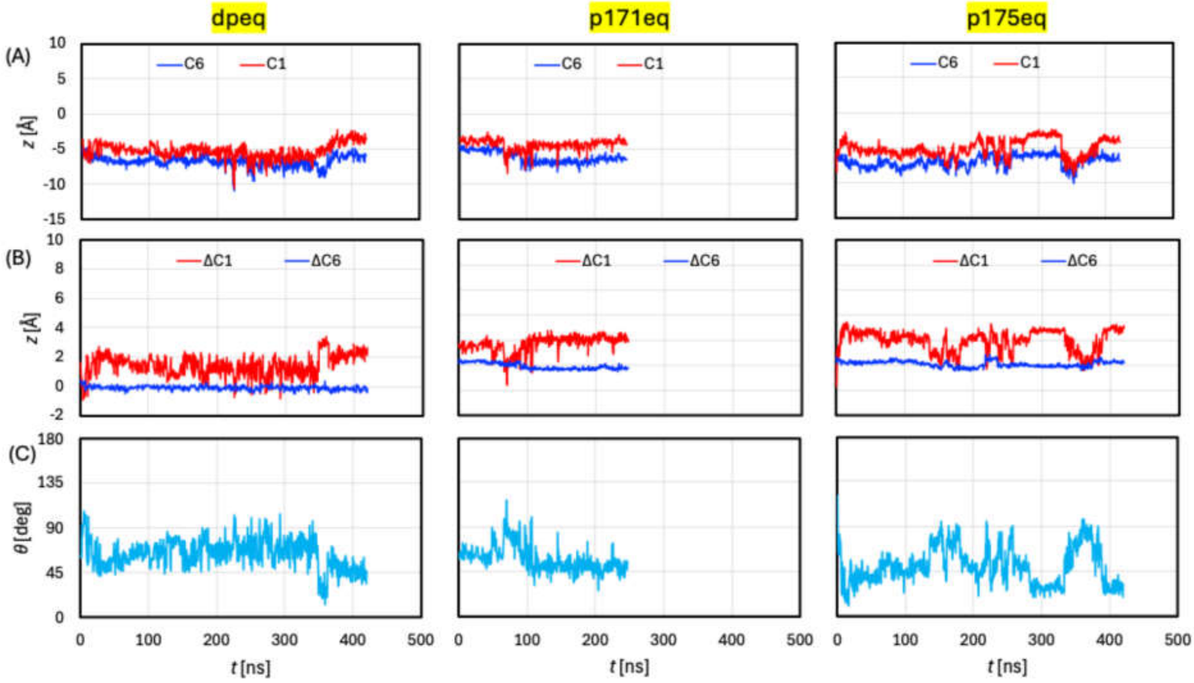

(**A**) *z* coordinate of the Neu5Ac C1 and C6 atoms. (**B**) Differences in *z* coordinates computed as $\Delta z(X) = z(X) - z(COM)$, where X = C1 or C6, and COM is the center of mass of the ligand. (**C**) The polar angle θ ($0° \le \theta \le 180°$) for the vector from COM to C1.

Figure S5. Backbone RMSD of individual residues averaged over time in SMD s1 trajectories.

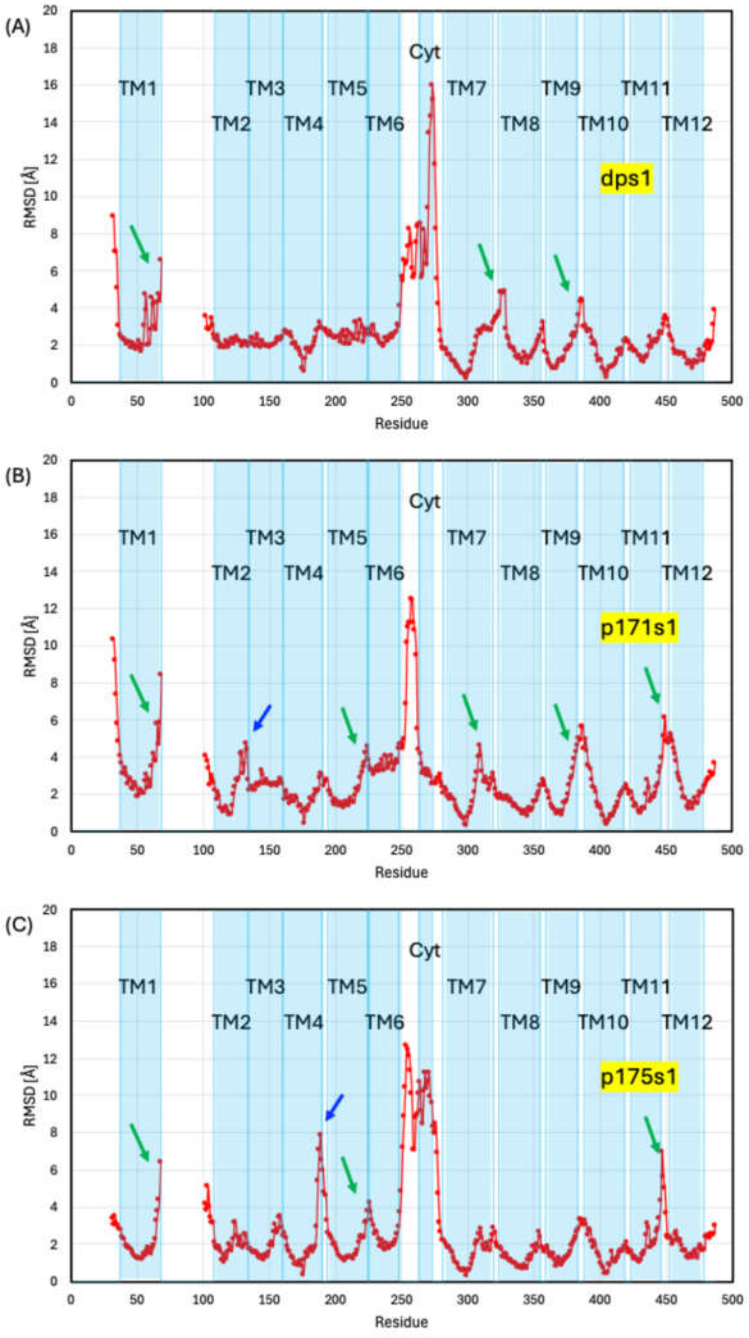

For models (**A**) dp, (**B**) p171, and (**C**) p175. The gap between TM1 and TM2 is due to the missing lumen loop in the experimental structure (PDB 8DWI). Cyt denotes the cytosol helix. Residues in TM and inter-TM loops (excluding the N-terminal and cytosol loops) with large RMSD (> 4 Å) are indicated by blue/green arrows if located on the cytosol/lumen side.

Figure S6. Ligand atom *z* coordinates over simulation time in SMD s1 trajectories.

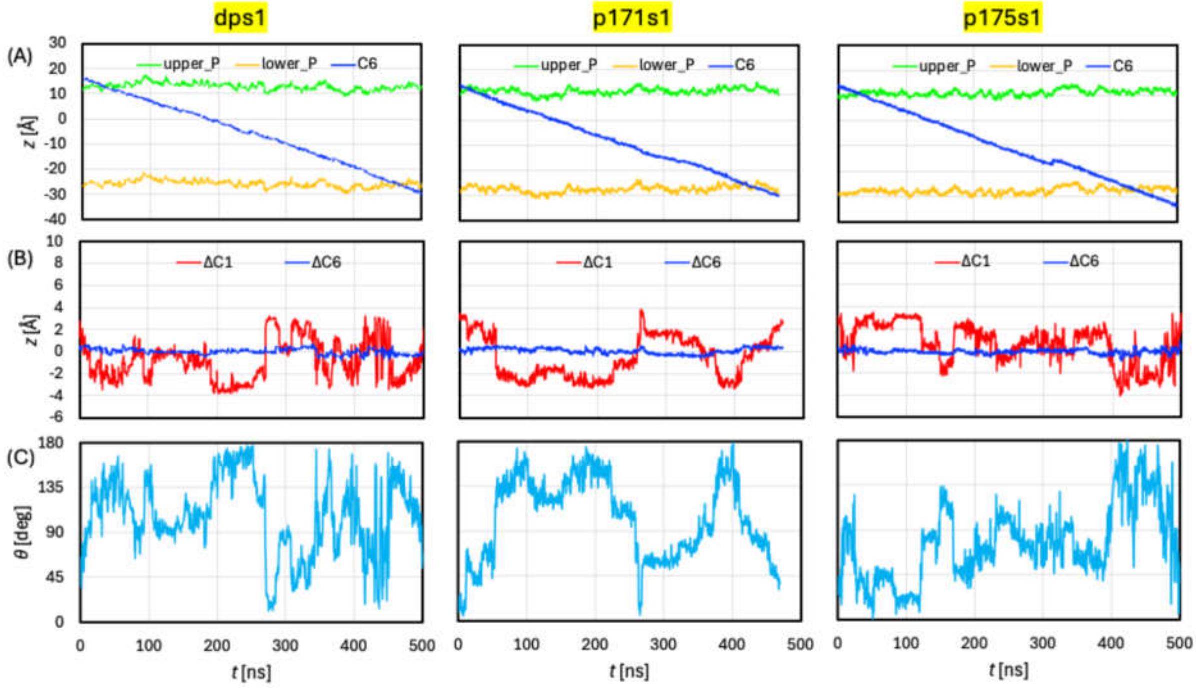

(**A**) *z* coordinate of the Neu5Ac C6 atom and of the average *z* coordinates of the lipid P atoms in the upper and lower leaflets, respectively. (**B**) Differences in *z* coordinates computed as $\Delta z(X) = z(X) - z(COM)$, where X = C1 or C6, and COM is the center of mass of the ligand. (**C**) The polar angle θ ($0° \leq \theta \leq 180°$) for the vector from COM to C1.

Figure S7. Overlays of representative geometries in SMD s1 trajectories and experimental structure.

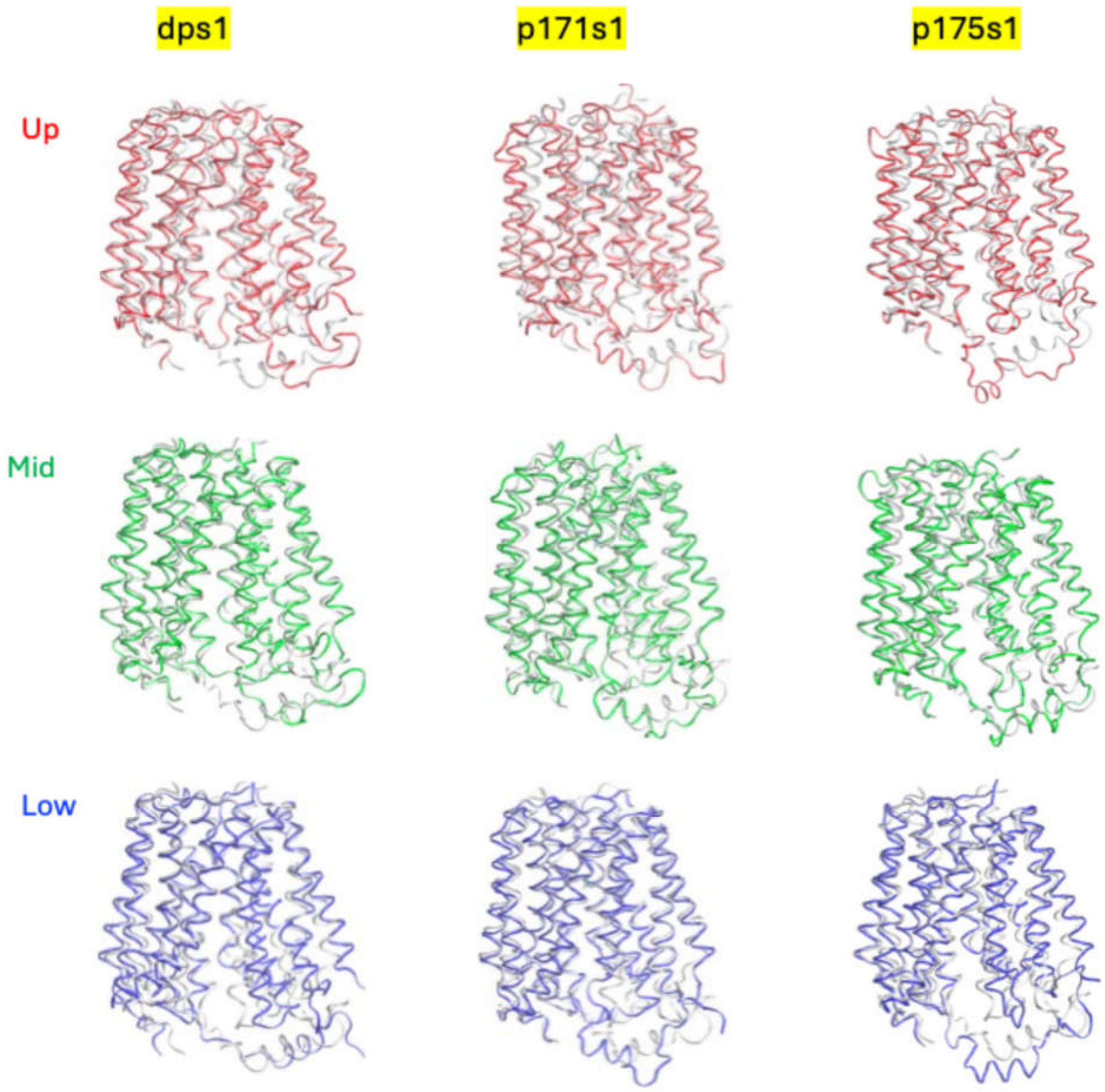

All proteins are shown as helixes and loops. The experimental structure (PDB 8DWI) is in gray color. The representative equilibrated geometries are in red for Up, green for Mid, and blue for Low, respectively.

Figure S8. Pores of representative geometries in SMD s1 trajectories and experimental structure.

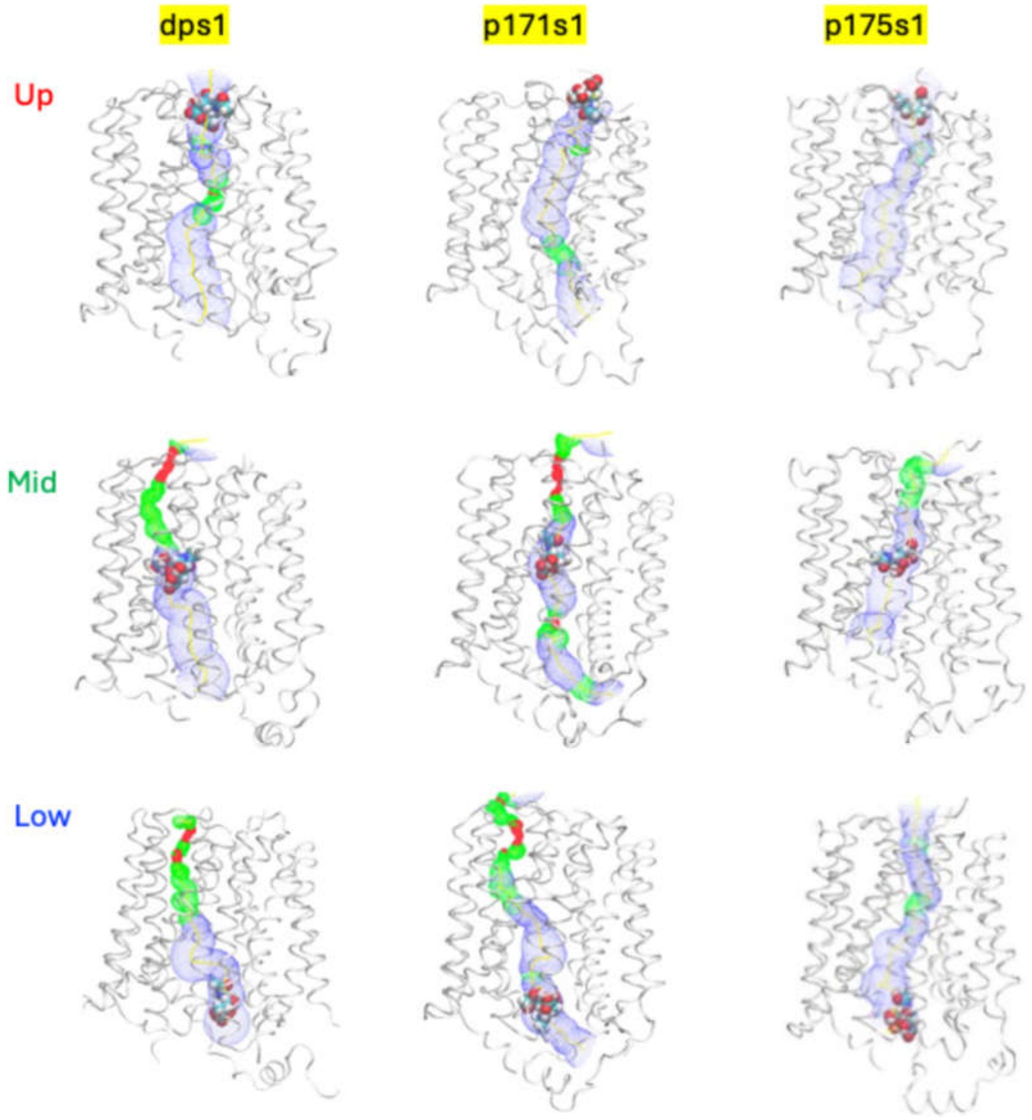

For the pore, red color indicates narrow sections (too tight for one water molecule), blue color wide sections (can accommodate two water molecules), and green color sections of medium-widths. The protein is displayed as helixes and loops in gray, and the ligand as spheres (color code: C, cyan; H, white; O, red, and N, blue).

Figure S9. Sialic acid transport model with enriched atomistic details.

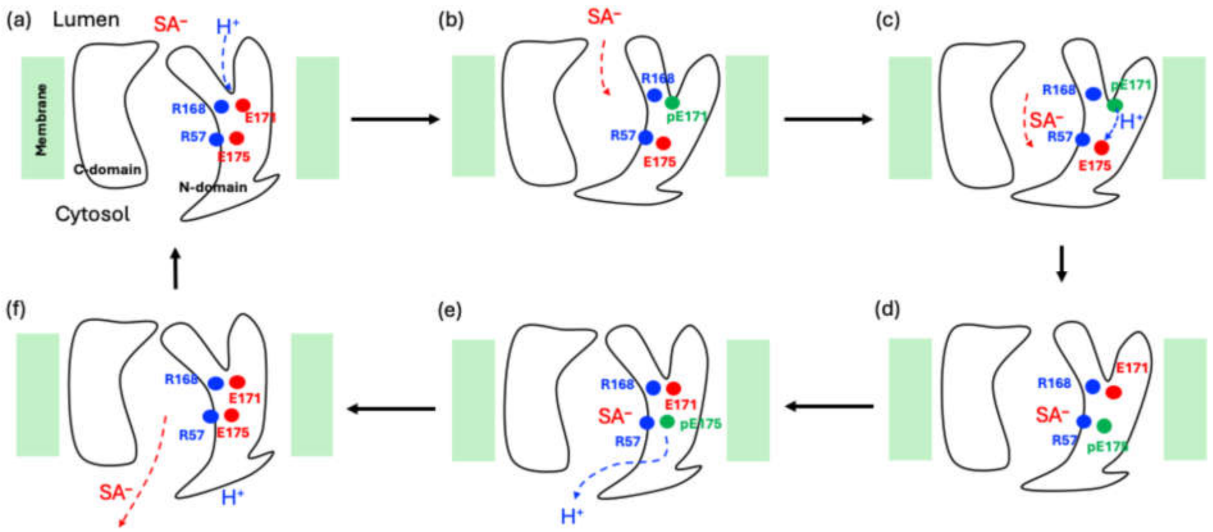

Figure S10. Additional plots for trajectory dps2.

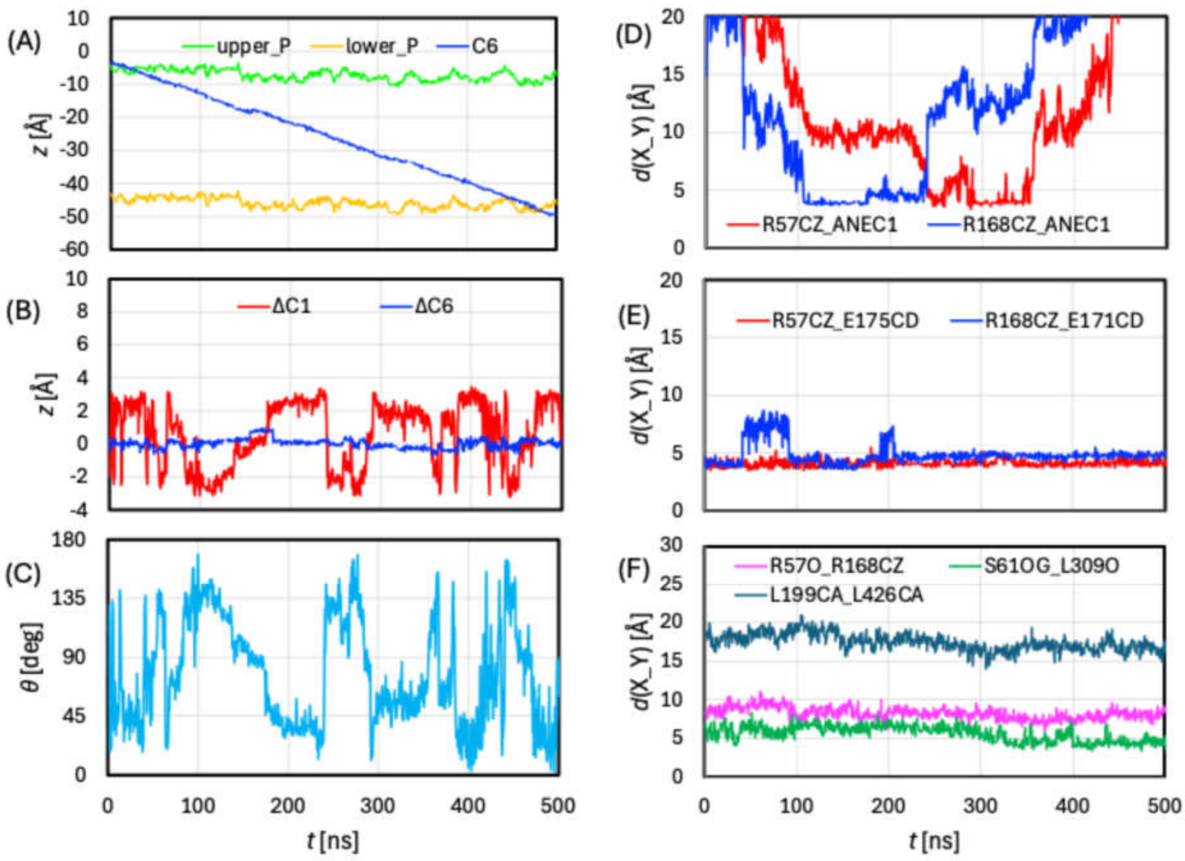

(**A**) *z* coordinate of the Neu5Ac C6 atom and of the average *z* coordinates of the lipid P atoms in the upper and lower leaflets, respectively. (**B**) Differences in *z* coordinates computed as $\Delta z(X) = z(X) - z(COM)$, where X = C1 or C6, and COM is the center of mass of the ligand. (**C**) The polar angle θ ($0° \leq \theta \leq 180°$) for the vector from COM to C1. (**D**) Distance from the Neu5Ac C1 atom to the Cζ atoms of R57 and R168. (**E**) Distances of the salt bridges between R57 and E175 and between R168 and E171, respectively. (**F**) Distance metrics for outward opening toward the lumen (R57O-R168Cζ and S61Oγ-L309O) and inward opening toward cytosol (L199Cα-L426Cα), respectively.

Figure S11. Pores of representative geometries in trajectory dps2.

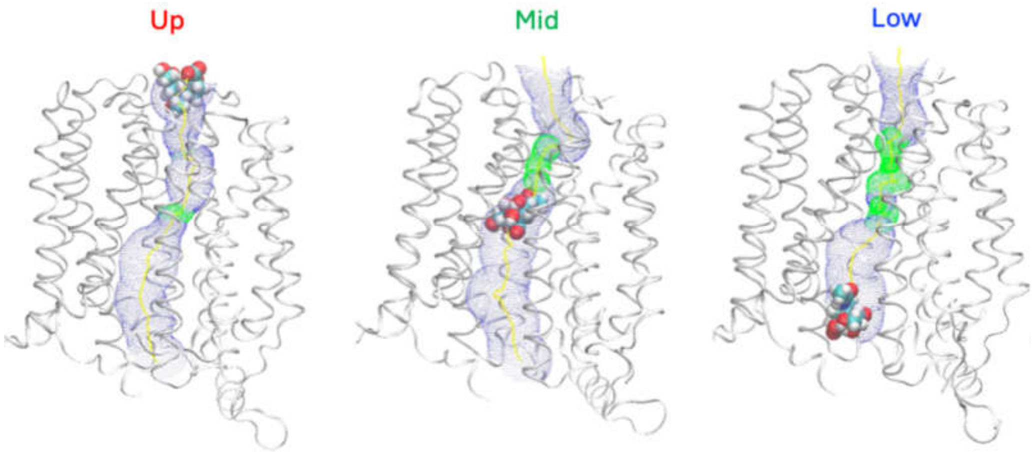

For the pore, red color indicates narrow sections (too tight for one water molecule), blue color wide sections (can accommodate two water molecules), and green color sections of medium-widths. The protein is displayed as helixes and loops in gray, and the ligand as spheres (color code: C, cyan; H, white; O, red, and N, blue).

Figure S12. Additional plots for trajectory dps3.

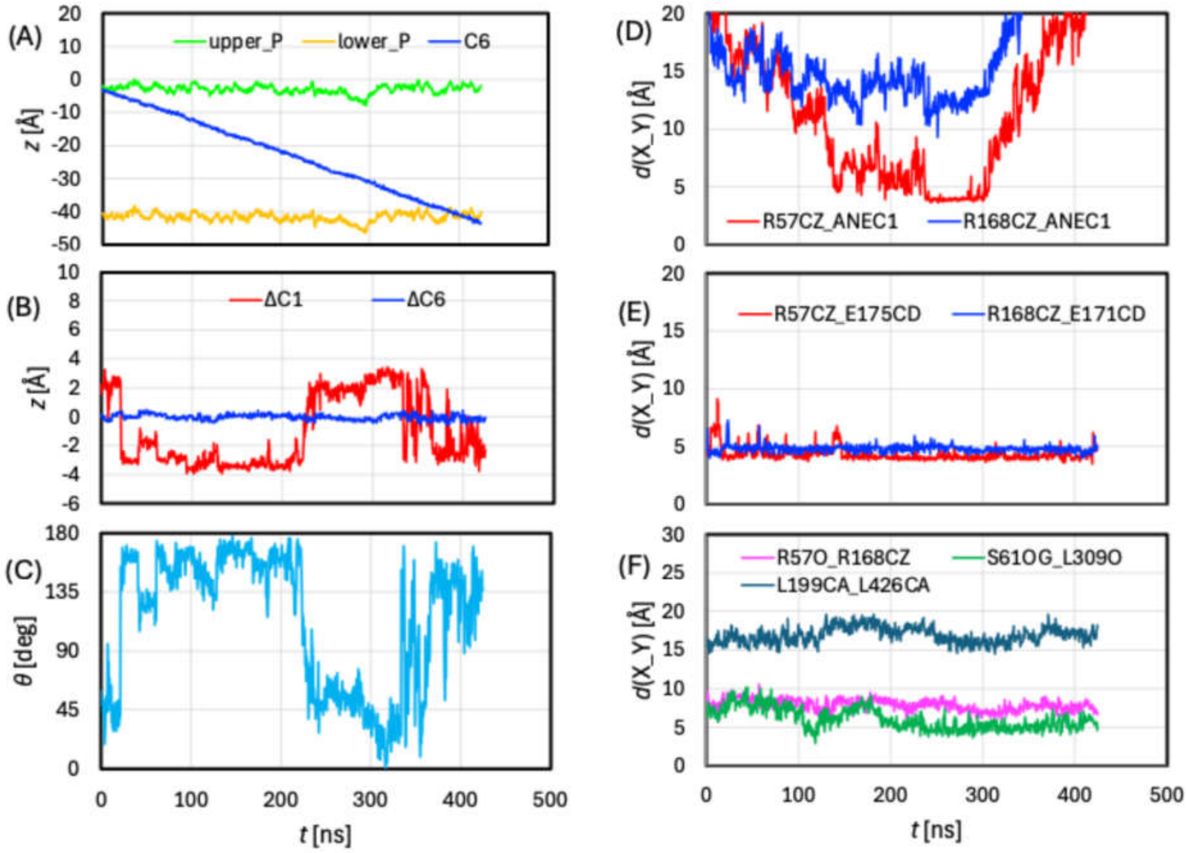

(**A**) *z* coordinate of the Neu5Ac C6 atom and of the average *z* coordinates of the lipid P atoms in the upper and lower leaflets, respectively. (**B**) Differences in *z* coordinates computed as $\Delta z(X) = z(X) - z(COM)$, where X = C1 or C6, and COM is the center of mass of the ligand. (**C**) The polar angle θ ($0° \leq \theta \leq 180°$) for the vector from COM to C1. (**D**) Distance from the Neu5Ac C1 atom to the Cζ atoms of R57 and R168. (**E**) Distances of the salt bridges between R57 and E175 and between R168 and E171, respectively. (**F**) Distance metrics for outward opening toward the lumen (R57O-R168Cζ and S61Oγ-L309O) and inward opening toward cytosol (L199Cα-L426Cα), respectively.

Figure S13. Pores of representative geometries in trajectory dps3.

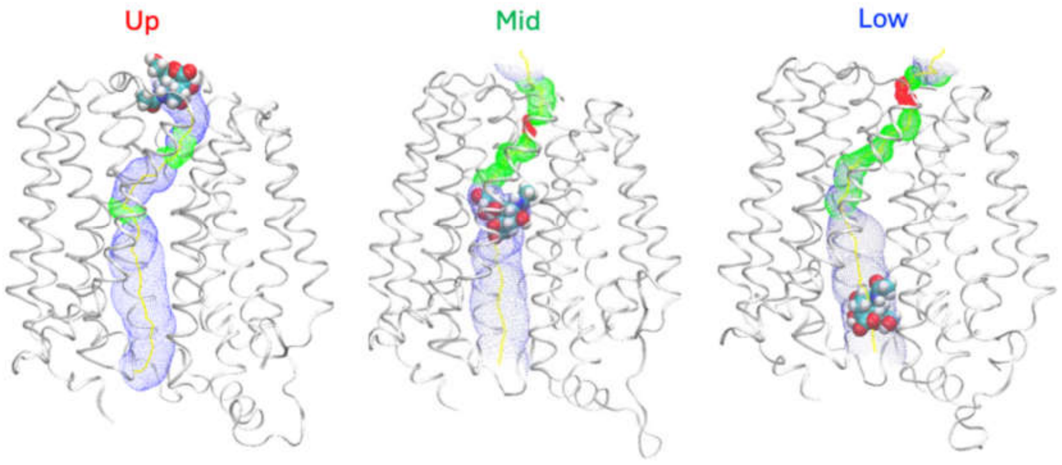

For the pore, red color indicates narrow sections (too tight for one water molecule), blue color wide sections (can accommodate two water molecules), and green color sections of medium-widths. The protein is displayed as helixes and loops in gray, and the ligand as spheres (color code: C, cyan; H, white; O, red, and N, blue).

Figure S14. Additional plots for trajectory p171s2.

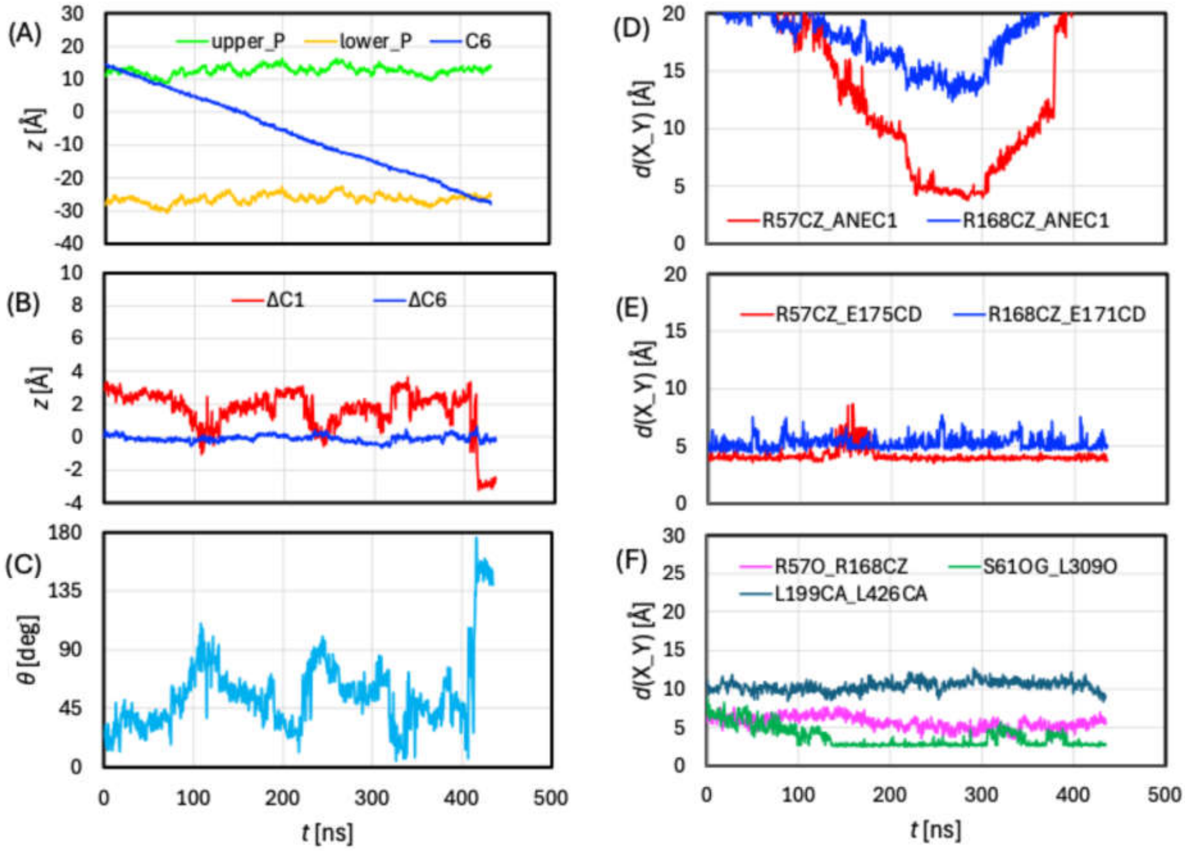

(**A**) *z* coordinate of the Neu5Ac C6 atom and of the average *z* coordinates of the lipid P atoms in the upper and lower leaflets, respectively. (**B**) Differences in *z* coordinates computed as $\Delta z(X) = z(X) - z(COM)$, where X = C1 or C6, and COM is the center of mass of the ligand. (**C**) The polar angle θ ($0° \le \theta \le 180°$) for the vector from COM to C1. (**D**) Distance from the Neu5Ac C1 atom to the Cζ atoms of R57 and R168. (**E**) Distances of the salt bridges between R57 and E175 and between R168 and E171, respectively. (**F**) Distance metrics for outward opening toward the lumen (R57O-R168Cζ and S61Oγ-L309O) and inward opening toward cytosol (L199Cα-L426Cα), respectively.

Figure S15. Pores of representative geometries in trajectory p171s2.

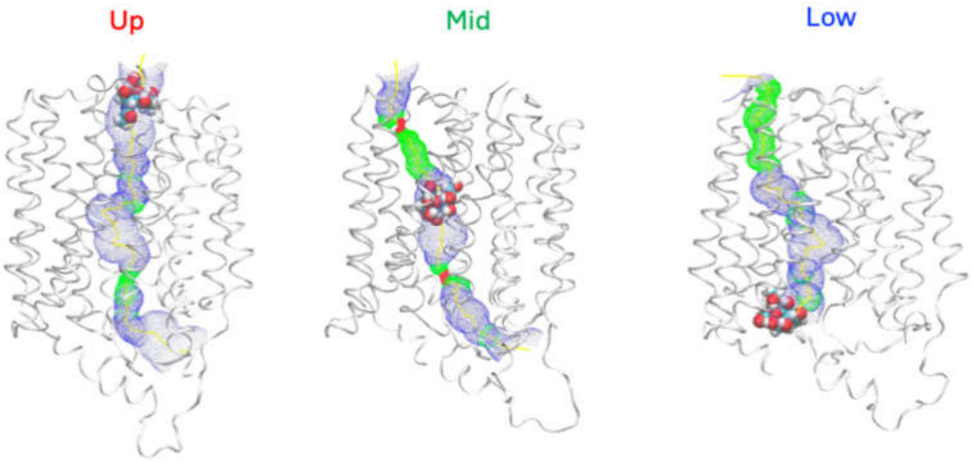

For the pore, red color indicates narrow sections (too tight for one water molecule), blue color wide sections (can accommodate two water molecules), and green color sections of medium-widths. The protein is displayed as helixes and loops in gray, and the ligand as spheres (color code: C, cyan; H, white; O, red, and N, blue).

Figure S16. Additional plots for trajectory p171s3.

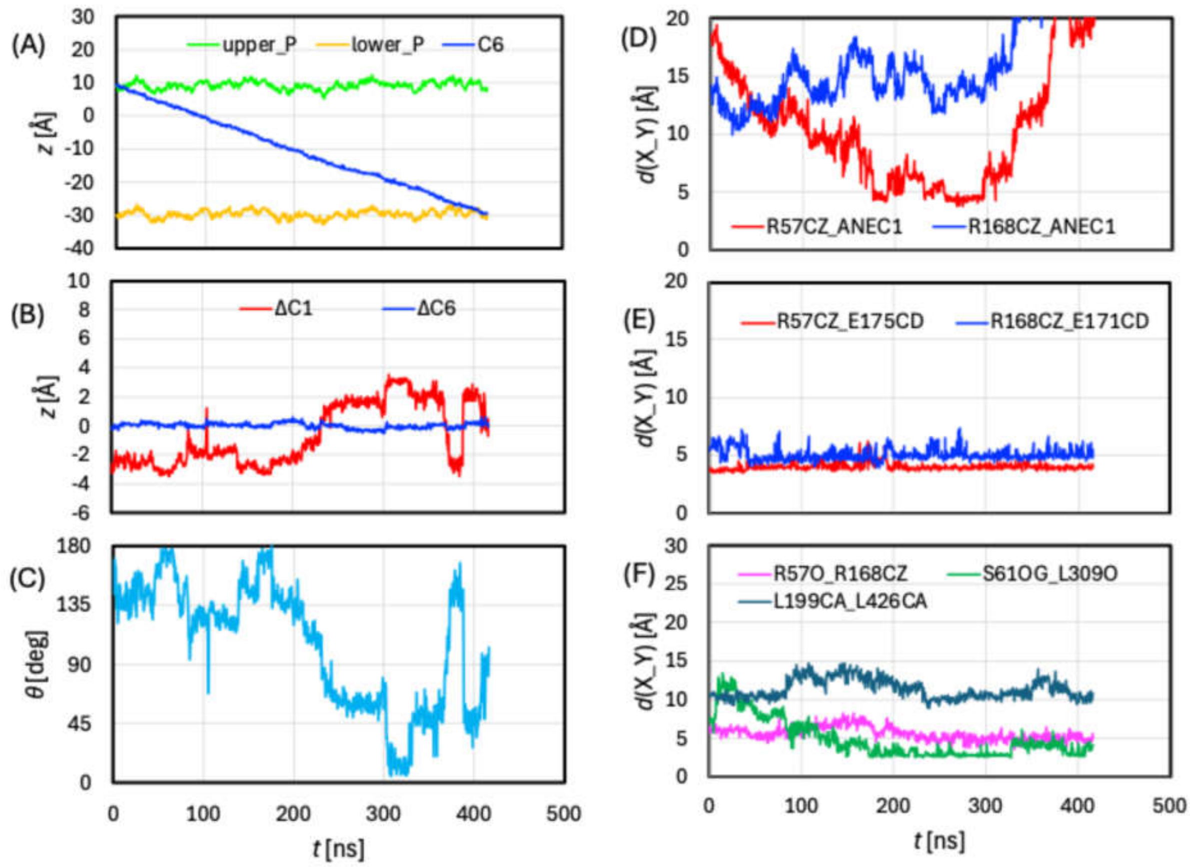

(**A**) *z* coordinate of the Neu5Ac C6 atom and of the average *z* coordinates of the lipid P atoms in the upper and lower leaflets, respectively. (**B**) Differences in *z* coordinates computed as $\Delta z(X) = z(X) - z(COM)$, where X = C1 or C6, and COM is the center of mass of the ligand. (**C**) The polar angle θ ($0° \leq \theta \leq 180°$) for the vector from COM to C1. (**D**) Distance from the Neu5Ac C1 atom to the Cζ atoms of R57 and R168. (**E**) Distances of the salt bridges between R57 and E175 and between R168 and E171, respectively. (**F**) Distance metrics for outward opening toward the lumen (R57O-R168Cζ and S61Oγ-L309O) and inward opening toward cytosol (L199Cα-L426Cα), respectively.

Figure S17. Pores of representative geometries in trajectory p171s3.

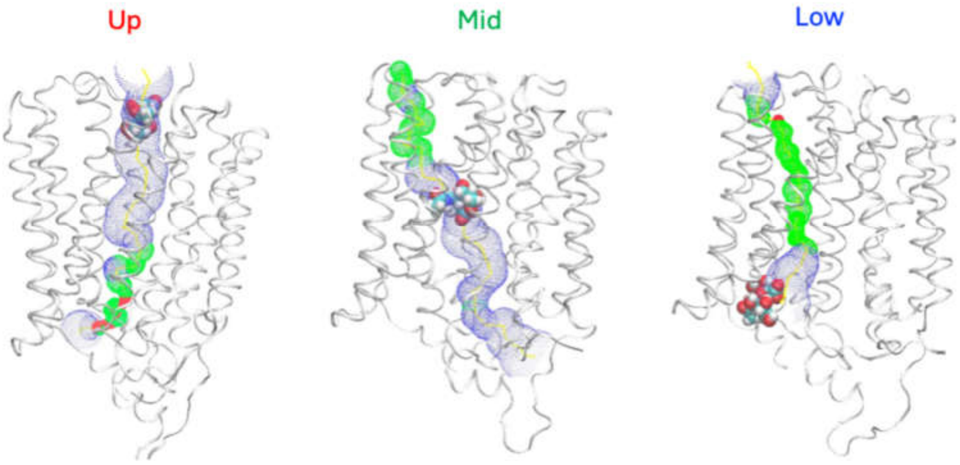

For the pore, red color indicates narrow sections (too tight for one water molecule), blue color wide sections (can accommodate two water molecules), and green color sections of medium-widths. The protein is displayed as helixes and loops in gray, and the ligand as spheres (color code: C, cyan; H, white; O, red, and N, blue).

Figure S18. Additional plots for trajectory p175s2.

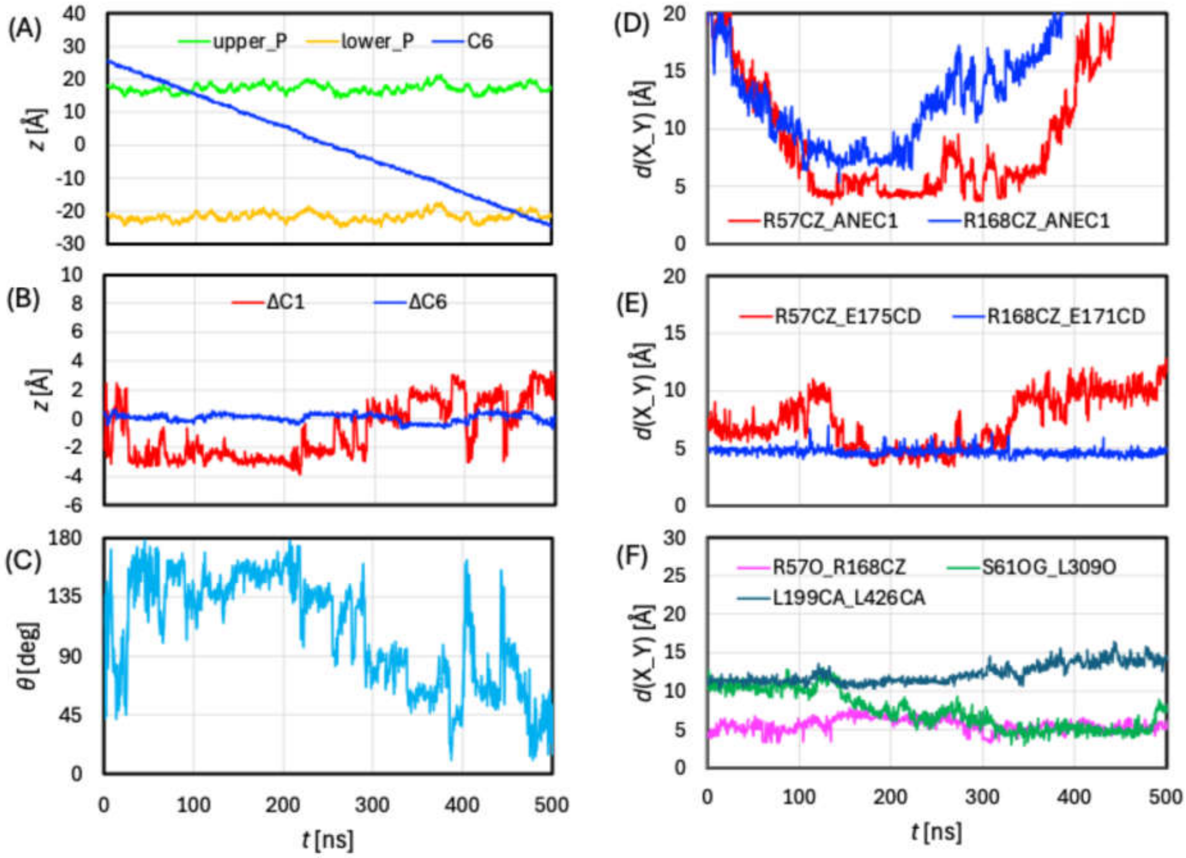

(**A**) *z* coordinate of the Neu5Ac C6 atom and of the average *z* coordinates of the lipid P atoms in the upper and lower leaflets, respectively. (**B**) Differences in *z* coordinates computed as $\Delta z(X) = z(X) - z(COM)$, where X = C1 or C6, and COM is the center of mass of the ligand. (**C**) The polar angle θ ($0° \leq \theta \leq 180°$) for the vector from COM to C1. (**D**) Distance from the Neu5Ac C1 atom to the Cζ atoms of R57 and R168. (**E**) Distances of the salt bridges between R57 and E175 and between R168 and E171, respectively. (**F**) Distance metrics for outward opening toward the lumen (R57O-R168Cζ and S61Oγ-L309O) and inward opening toward cytosol (L199Cα-L426Cα), respectively.

Figure S19. Pores of representative geometries in trajectory p175s2.

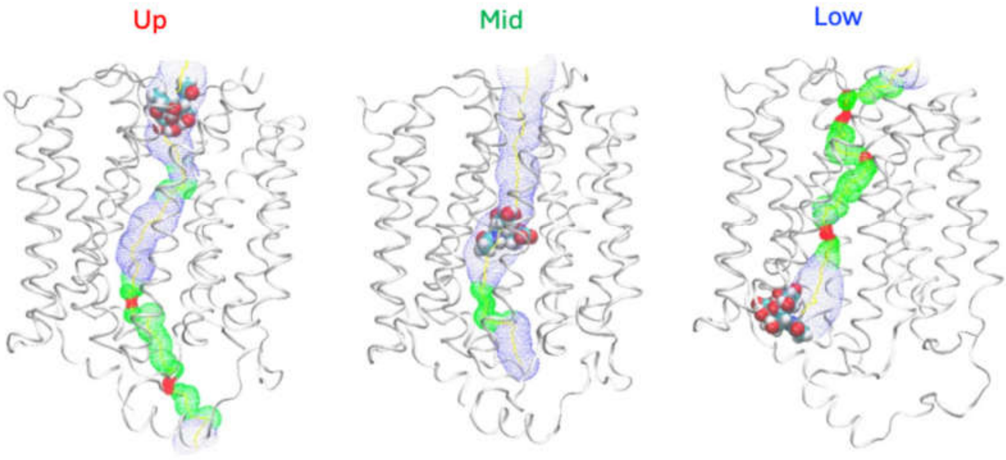

For the pore, red color indicates narrow sections (too tight for one water molecule), blue color wide sections (can accommodate two water molecules), and green color sections of medium-widths. The protein is displayed as helixes and loops in gray, and the ligand as spheres (color code: C, cyan; H, white; O, red, and N, blue).

Figure S20. Additional plots for trajectory p175s3.

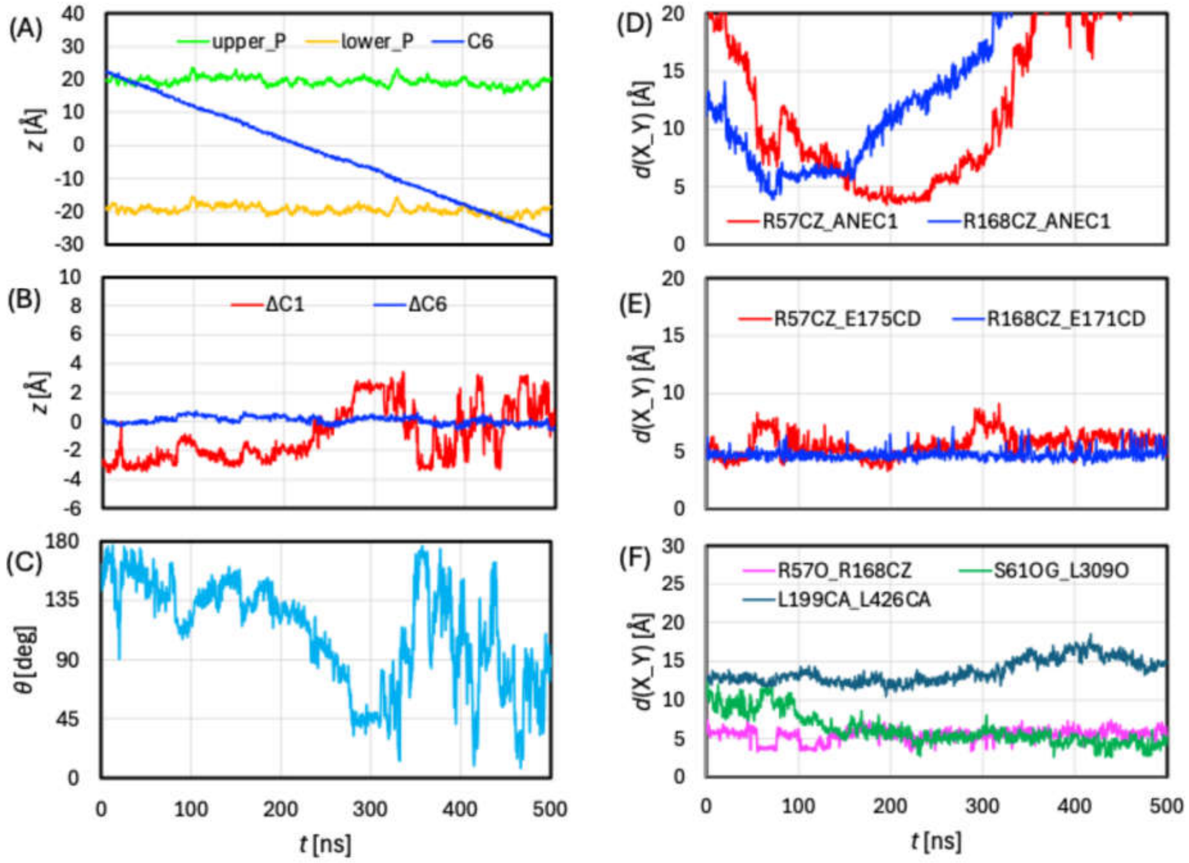

(**A**) *z* coordinate of the Neu5Ac C6 atom and of the average *z* coordinates of the lipid P atoms in the upper and lower leaflets, respectively. (**B**) Differences in *z* coordinates computed as $\Delta z(X) = z(X) - z(COM)$, where X = C1 or C6, and COM is the center of mass of the ligand. (**C**) The polar angle θ ($0° \leq \theta \leq 180°$) for the vector from COM to C1. (**D**) Distance from the Neu5Ac C1 atom to the Cζ atoms of R57 and R168. (**E**) Distances of the salt bridges between R57 and E175 and between R168 and E171, respectively. (**F**) Distance metrics for outward opening toward the lumen (R57O-R168Cζ and S61Oγ-L309O) and inward opening toward cytosol (L199Cα-L426Cα), respectively.

Figure S21. Pores of representative geometries in trajectory p175s3.

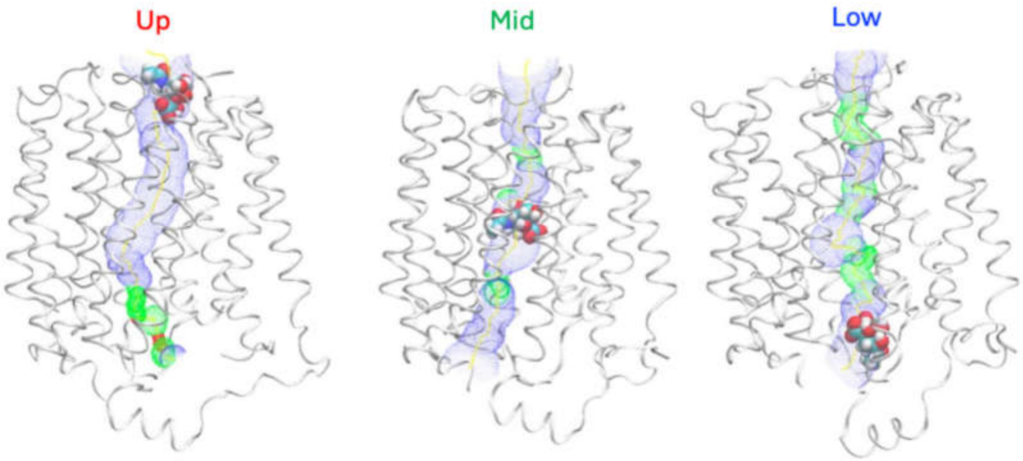

For the pore, red color indicates narrow sections (too tight for one water molecule), blue color wide sections (can accommodate two water molecules), and green color sections of medium-widths. The protein is displayed as helixes and loops in gray, and the ligand as spheres (color code: C, cyan; H, white; O, red, and N, blue).

Figure S22. Pulling force against time in SMD simulations.

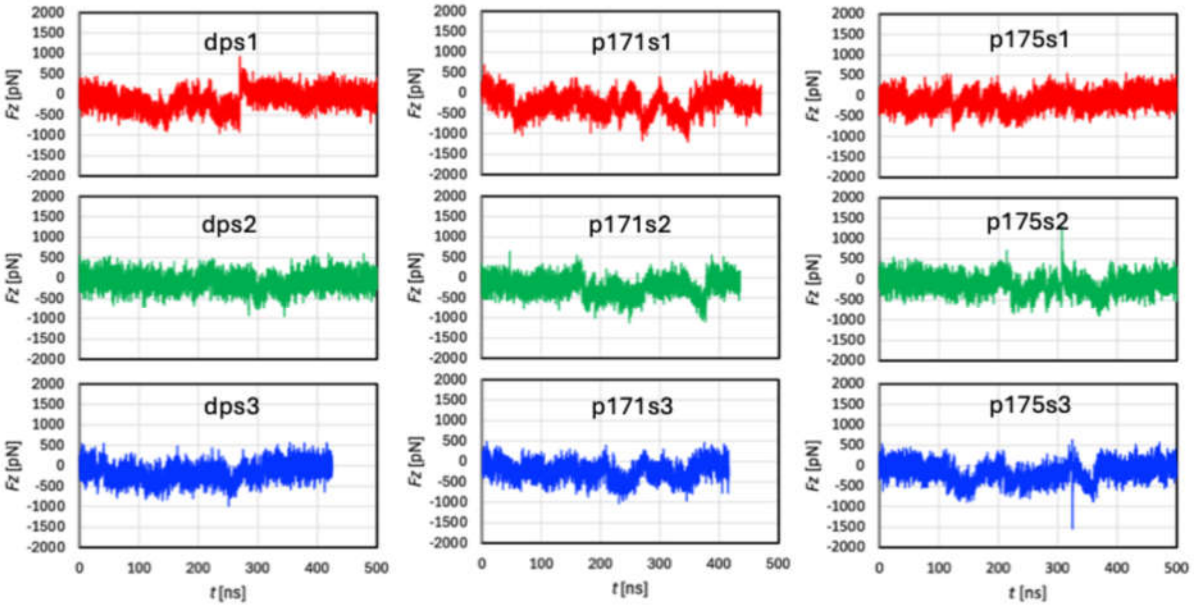

Figure S23. Accumulated pulling work against time in SMD simulations.

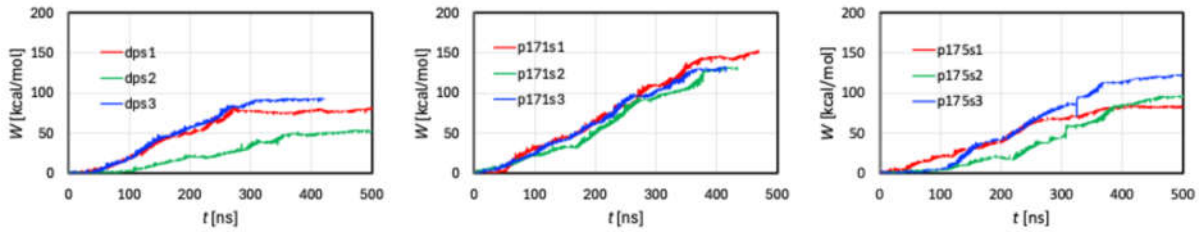

Supplement: Supplementary file 1 [file ijms-27-04629-s001.zip › ijms-4311890-supplementary.pdf]
